# Engineered T cells secreting αB7-H3-αCD3 bispecific engagers for enhanced anti-tumor activity against B7-H3 positive multiple myeloma: a novel therapeutic approach

**DOI:** 10.1186/s12967-024-05923-z

**Published:** 2025-01-13

**Authors:** Punchita Rujirachaivej, Teerapong Siriboonpiputtana, Kornkan Choomee, Kamonlapat Supimon, Thanich Sangsuwannukul, Pucharee Songprakhon, Krissada Natungnuy, Piriya Luangwattananun, Pornpimon Yuti, Mutita Junking, Pa-thai Yenchitsomanus

**Affiliations:** 1 Graduate Program in Clinical Pathology, Department of Pathology, Faculty of Medicine Ramathibodi Hospital, Mahidol University, Bangkok, Thailand; 2 Department of Pathology, Faculty of Medicine Ramathibodi Hospital, Mahidol University, Bangkok, Thailand; 3 Siriraj Center of Research Excellence for Cancer Immunotherapy (SiCORE-CIT), Faculty of Medicine Siriraj Hospital, Mahidol University, Bangkok, Thailand; 4 Division of Molecular Medicine, Research Department, Faculty of Medicine Siriraj Hospital, Mahidol University, Bangkok, Thailand; 5 Department of Molecular Medicine, Mayo Clinic Rochester, Rochester, MN USA

**Keywords:** Multiple myeloma, Bispecific T-cell engager, BITE, T cells secreting BITE, ENG T cells, B7-H3, CD276

## Abstract

**Background:**

Multiple myeloma (MM) is an incurable plasma cell malignancy with increasing global incidence. Chimeric antigen receptor (CAR) T-cell therapy targeting BCMA has shown efficacy in relapsed or refractory MM, but it faces resistance due to antigen loss and the tumor microenvironment. Bispecific T-cell engaging (BITE) antibodies also encounter clinical challenges, including short half-lives requiring continuous infusion and potential toxicities.

**Methods:**

To address these issues, we developed a lentiviral system to engineer T cells that secrete αB7-H3-αCD3 bispecific engager molecules (αB7-H3-αCD3 ENG-T cells). We evaluated their effectiveness against MM cells with varying B7-H3 expression levels, from B7-H3^neg^ to B7-H3^high^.

**Results:**

The αB7-H3-αCD3 ENG-T cells demonstrated significant anti-tumor activity against MM cell lines expressing B7-H3. SupT-1 cells (B7-H3^neg^) served as controls and exhibited minimal cytotoxicity from αB7-H3-αCD3 ENG T cells. In contrast, these engineered T cells showed dose-dependent killing of B7-H3-expressing MM cells: NCI-H929 (B7-H3^low^), L-363 (B7-H3^medium^), and KMS-12-PE (B7-H3^high^). For NCI-H929 cells, cytotoxicity reached 38.5 ± 7.4% (*p* = 0.0212) and 54.0 ± 9.2% (*p* = 0.0317) at effector-to-target (E:T) ratios of 5:1 and 10:1, respectively. Against L-363 cells, cytotoxicity was 56.6 ± 3.2% (*p* < 0.0001) and 71.4 ± 5.2% (*p* = 0.0002) at E:T ratios of 5:1 and 10:1, respectively. For KMS-12-PE cells, significant cytotoxic effects were observed even at an E:T ratio of 1:1, with 27.2 ± 3.7% (*p* = 0.0004), 44.4 ± 3.7% (*p* < 0.0001), and 68.6 ± 9.2% (*p* = 0.0004) cytotoxicity at E:T ratios of 1:1, 5:1, and 10:1, respectively.

**Conclusions:**

These results indicate that αB7-H3-αCD3 ENG T cells could be a promising therapy for B7-H3-positive MM. They may enhance current MM treatments and improve overall outcomes. Additional preclinical and clinical research is required to fully assess their therapeutic potential.

**Supplementary Information:**

The online version contains supplementary material available at 10.1186/s12967-024-05923-z.

## Background

Multiple myeloma (MM) is an incurable hematologic malignancy characterized by the clonal expansion of dysfunctional plasma cells in the bone marrow, resulting in approximately 106,000 deaths annually worldwide [1, 2]. Current standard therapies for MM typically involve a regimen comprising chemotherapy, steroids, and autologous stem cell transplantation (ASCT) [3]. Noteworthy progress has been achieved in MM treatment with the introduction of novel agents such as proteasome inhibitors (PIs), immunomodulatory drugs (IMiDs), and monoclonal antibodies (mAbs), thus enhancing the life expectancy of MM patients [4]. However, drug resistance remains a significant challenge, particularly in relapsed/refractory MM (RRMM) cases [5, 6]. Immunotherapeutic strategies, such as monoclonal antibodies targeting CD38 (e.g., daratumumab) and SLAMF7 (e.g., elotuzumab), have emerged as promising treatments for multiple myeloma. These strategies target MM cells by inducing antibody-dependent cellular cytotoxicity (ADCC) and apoptosis [7, 8]. Additionally, cellular immunotherapies such as chimeric antigen receptor (CAR) T-cell therapy have demonstrated efficacy in treating RRMM [9–11]. FDA-approved CAR T-cell therapies targeting B-cell maturation antigen (BCMA), including idecabtagene vicleucel (ide-cel) and ciltacabtagene autoleucel (cilta-cel), have exhibited effectiveness in heavily pretreated RRMM patients [10–12]. CAR T cell therapies directed against BCMA have elicited notable responses in the treatment of MM. Nonetheless, significant challenges persist, encompassing cytokine release syndrome (CRS), neurotoxicity, and the emergence of therapeutic resistance [13, 14].

The immunosuppressive tumor microenvironment plays a pivotal role in MM pathobiology and treatment resistance mechanisms [15]. Consequently, several immunotherapeutic approaches have been developed to overcome these challenges [16]. Among them, bispecific T-cell engagers (BITEs) have gained regulatory approval and are employed in the treatment of hematological malignancies [17–22]. BITE are recombinant proteins comprising antigen-binding antibodies targeting tumor-associated antigens and T cell-specific CD3 domains, facilitating T cell activation and tumor cell killing [17]. Blinatumomab, the first FDA-approved BITE (CD19/CD3), was introduced in 2014 [18, 23] with subsequent approvals anticipated for Mosunetuzumab and Epcoritamab (CD20/CD3) [19, 20], and Teclistamab (BCMA/CD3) [21]. Despite their efficacy, BITEs exhibit a short half-life, necessitating continuous systemic infusion, potentially leading to toxicities and limited biodistribution [22, 24].

One promising approach to address these limitations involves genetic modification and adoptive transfer of T cells that secrete bispecific engager molecules (ENG T cells). ENG T cells have shown potential in preclinical models for solid tumors [25, 26], although data for MM are currently lacking. In this study, we present the generation of engineered T cells (ENG T cells) designed to target the tumor-associated antigen B7 Homolog 3 (B7-H3 or CD276) in conjunction with CD3. B7-H3, a type I transmembrane protein consisting of 316 amino acids [27], is markedly upregulated in diverse malignancies, including MM, while its expression remains minimal in normal tissues [28, 29], thereby rendering it a promising therapeutic target [30]. Reduced B7-H3 expression in MM correlates with enhanced progression-free survival, whereas its upregulation is implicated in heightened drug resistance and tumor advancement [24]. This study investigates the anti-leukemia activities of αB7-H3-αCD3 ENG T cells specific for B7-H3-positive MM cell lines. Our findings demonstrate their antigen-dependent activation, tumor cell eradication, and bystander T-cell recruitment, highlighting their potential as a novel therapeutic strategy for MM.

## Materials and methods

### Cell culture

The human MM cell lines NCI-H929, L-363, and KMS-12-PE were generously provided by Professor Seiji Okada from the Center for AIDS Research and Graduate School of Medical Sciences, Division of Hematopoiesis, Kumamoto University, Japan. SupT-1 cell lines, derived from human T cell lymphoblastic lymphoma, were procured from the American Type Culture Collection (ATCC) (Cat# CRL-1942, RRID: CVCL_1714, Manassas, VA, USA). NCI-H929, L-363, KMS-12-PE, and SupT-1 cells were cultured in Roswell Park Memorial Institute (RPMI)−1640 medium (Gibco; Invitrogen Corporation, Carlsbad, CA, USA) supplemented with 10% heat-inactivated fetal bovine serum (FBS) (Gibco; Invitrogen) and 100 μg/mL of penicillin/streptomycin (Sigma-Aldrich Corporation, St. Louis, MO, USA). Lenti-X™ human embryonic kidney (HEK) 293 T cell lines (Takara Bio, Inc., Shiga, Japan) and HEK293T cell lines were cultured in Dulbecco’s Modified Eagle Medium (DMEM) (Gibco; Thermo Fisher Scientific, Waltham, MA, USA) containing 10% FBS and 100 μg/mL of penicillin/streptomycin. All cell lines were maintained at 37 °C in a 5% CO_2_ atmosphere.

### Construction of αB7-H3-αCD3 bispecific T-cell engager

The bispecific T-cell engager (BITE) construct αB7-H3-αCD3, targeting B7-H3 and CD3, was designed to incorporate a signal peptide followed by the codon-optimized cDNA sequences encoding αB7-H3, derived from a humanized version of the hu8H9 single-chain variable fragment (scFv) [31], and αCD3 scFv obtained from OKT3, along with a Gly4Ser linker and a Myc-tag. Synthesis of the αB7-H3-αCD3 BITE construct was conducted by Synbio Technologies, U.S.A. Subsequently, this construct was cloned into the polyclonal site of the pCDH-CMV-MCS-EF1-copGFP lentiviral vector (System Biosciences, Palo Alto, USA) using EcoRI and NotI restriction enzymes, resulting in the generation of the pCDH-CMV.αB7-H3-αCD3-BITE.EF1-copGFP vector.

### Immunoblotting analysis

To assess the expression and secretion of αB7-H3-αCD3 bispecific T-cell engagers (BITEs), stable cell lines expressing the αB7-H3-αCD3 BITE were established utilizing the lentiviral vector system. Lentiviral particles carrying the αB7-H3-αCD3 BITE constructs were generated in HEK293T cells. Subsequently, these lentiviral particles containing the αB7-H3-αCD3 BITE were transduced into HEK293T cells to generate stable cell lines expressing the αB7-H3-αCD3 BITE. These stable cell lines were cultured in T75 flask with DMEM media (Gibco; Thermo Fisher Scientific) for 72 h to facilitate the collection of the secreted BITE protein in the culture supernatant. Both the HEK293T cells stably expressing αB7-H3-αCD3 BITE and their culture supernatant were harvested. Whole-cell lysates were prepared using 1% NP-40 lysis buffer. The extracted proteins from both the lysates and culture supernatants were separated by 12% sodium dodecyl sulfate–polyacrylamide gel electrophoresis (SDS-PAGE) and subsequently transferred onto a nitrocellulose membrane. Following transfer, the membrane was blocked with 5% skim milk in Tris-buffered saline and 0.1% Tween-20 (TBST), followed by probing with an anti-cMyc tag antibody (Clone 9E10, Santa Cruz Biotechnology, CA, USA) and a rabbit anti-mouse HRP-conjugated secondary antibody. Internal control for whole-cell lysates was performed using anti-human GAPDH antibodies (Clone 0411; Santa Cruz Biotechnology). The concentration of the BITE protein in the culture supernatant was assessed via immunoblotting and semiquantitative analysis, with a comparison made to know A20/αCD3 BITE concentrations from a separate research study [32].

### Binding assay

The capacity of αB7-H3-αCD3 BITEs to interact with B7-H3 and CD3 proteins on the cell surface was assessed using flow cytometry. The binding proficiency of αB7-H3-αCD3 BITEs to B7-H3 and CD3 proteins was examined in KMS-12-PE cells and phytohemagglutinin-L (PHA-L) activated T cells, respectively. Briefly, KMS-12-PE cells or PHA-L activated T cells were treated with 100 μL of culture supernatant obtained from HEK293T cells stably expressing αB7-H3-αCD3 BITEs for 1 h at 4 °C. Following washing steps, KMS-12-PE cells or PHA-L activated T cells were incubated with anti-Myc-fluorescein isothiocyanate (FITC) antibody (Clone ab1394; Abcam, Cambridge, UK) for 30 min at 4 °C. Subsequently, the fluorescence signal from the monoclonal antibody was detected by flow cytometry, and compared to that of untransduced controls.

### Lentiviral production

To generate lentiviral particles, the Lenti-X™ 293 T cells were co-transfected with three plasmids: the transfer plasmid (αB7-H3-αCD3 BITE), the structural plasmid (psPAX2) containing HIV gag-pol genes, and the envelope plasmid (pMD2.G) containing the VSV-G gene, at a ratio of 5:3.5:1. This was done using the calcium phosphate precipitation method. After 48- and 72-h post-transfection, culture supernatants containing lentiviral particles were harvested and filtered through a 0.45 μm membrane to eliminate cellular debris. The viral particles were subsequently concentrated via high-speed centrifugation at 20,000×*g* for 180 min at 4 °C and stored at 4 °C for future investigations.

### T cell isolation and transduction

Peripheral blood mononuclear cells (PBMCs) were isolated from healthy donors via density gradient centrifugation using Lymphocyte Separation Medium (Corning, Inc., New York, NY, USA). Subsequently, PBMCs were seeded in culture dishes to facilitate the adhesion of undesirable monocytes in AIM-V medium (Gibco, Waltham, MA, USA) supplemented with 5% human AB serum (Sigma-Aldrich). T cells were activated for 72 h with phytohemagglutinin-L (PHA-L) at a concentration of 5 μg/mL (Roche, Basel, Switzerland), along with recombinant human interleukin (rhIL)−2 (10 ng/mL), rhIL-7 (5 ng/mL), and rhIL-15 (20 mg/mL) (Immunotools, Friesoythe, Germany). Following activation with PHA-L, T cells were transduced with lentiviruses and subjected to spinoculation at 1200*g* for 90 min at 32 °C in the presence of 10 μg/mL protamine sulfate (Sigma-Aldrich). Transduced T cells were then cultured in a medium supplemented with rhIL-2 (10 ng/mL), rhIL-7 (5 ng/mL), and rhIL-15 (20 mg/mL). The expression of αB7-H3-αCD3 BITEs on transduced T cells was evaluated using flow cytometry 48 or 72 h after post-transduction.

### Flow cytometry

To assess B7-H3 surface expression, the cell lines underwent a sequential procedure: initial washing followed by staining with a primary B7-H3 antibody (F-11) sourced from Santa Cruz Biotechnology, Inc. (catalog number sc-376769), conducted at 4 °C for 60 min. Subsequently, they were rinsed with 2% FBS/PBS and incubated with an Alexa Fluor 488 conjugated secondary antibody for 30 min. Evaluation of HEK293T cells, constitutively expressing αB7-H3-αCD3 BITE, and determination of the transduction efficiency of αB7-H3-αCD3 ENG T cells were performed by detecting CopGFP, integrated within the construct, using a fluorescent filter set at FITC. The T-cell phenotype characterization involved staining with various antibodies, including anti-CD3-APC (Clone UCHT-1), anti-CD3-PerCP (Clone UCHT-1), anti-CD4-PE (Clone MEM-241), anti-CD8-APC (Clone UCHT-4), anti-CD16-APC (Clone 3G8), anti-CD19 APC (Clone LT19), and CD62L-PE (Clone HI62L), all procured from ImmunoTools GmbH (Friesoythe, Germany). Additionally, anti-CD56-PE (Clone AB_2563925) and anti-45RA-PE-Cyanine7 (Clone HI100) were acquired from BioLegend (CA, USA) and eBioscience (San Diego, CA), respectively. Assessment of T cell exhaustion was conducted via the following antibodies: anti-PD-1-PE (Clone EH12.2H7), anti-LAG3-PE (Clone C9B7W), and anti-TIM3-PE (Clone F38-2E2), obtained from BioLegend. Following a 30-min incubation with monoclonal antibodies in the dark at 4 °C, cells underwent two washes before analysis on a BD Accuri™ C6 Plus Flow Cytometer (BD Biosciences, Franklin Lakes, NJ, USA). Flow cytometry data analysis was performed using FlowJo 10.0 software (FlowJo LLC, Ashland, OR USA).

### Immunofluorescence assay (IFA)

Cell suspensions of SupT-1, NCI-H929, L-363, and KMS-12-PE cell lines were prepared and spread onto slides, followed by fixation with 100% methanol at − 20 °C for 10 min, and subsequent washing with 1 × phosphate-buffered saline (PBS). Following fixation, the cells underwent incubation with B7-H3 Antibody (F-11) (Santa Cruz Biotechnology, Inc., catalog number sc-376769) at a dilution of 1:100 for 60 min at room temperature (RT), or with an isotype control (anti-mouse IgG2a antibody produced in goat). Subsequently, the cells were incubated with an Alexa Fluor 488-conjugated secondary antibody (dilution 1:1000) containing Hoechst 33342 (dilution 1:5000) at RT in darkness for 60 min. Post-incubation, the cells were washed and observed, and images were captured using confocal microscopy. The percentage of HEK293T cells stably expressing αB7-H3-αCD3 BITE was evaluated by assessing copGFP, which was tagged in the construct, and images were captured using confocal microscopy.

### Flow cytometry-based cytotoxicity and proliferation activity

The target cells were labeled with 0.75 µM of CellTracker™ Orange CMRA Dye (Thermo Fisher Scientific). Co-culture assays were conducted by incubating αB7-H3-αCD3 ENG T cells and un-transduced T cells (UTD T) with CMRA-labeled target cells for 24 h at effector to target (E:T) ratios of 1:1, 5:1, and 10:1. Subsequent to co-culturing, counting beads (123count™ eBeads Counting Beads; Thermo Fisher Scientific) were introduced to each sample to facilitate the quantification of the absolute number of target cells through flow cytometry analysis, adhering to the manufacturer's protocol. The percentage of tumor cell cytotoxicity was calculated using the formula: (1 − (target cells in each condition/target cells alone at the indicated time)) × 100. To evaluate proliferation activity, αB7-H3-αCD3 ENG T cells and UTD T cells were labeled with 5 μM CellTrace™Far Red dye (Invitrogen) and co-cultured with target cells at an E:T ratio of 5:1 in the absence of exogenous cytokines. Following 5 days of co-cultivation, CellTrace™Far Red (CTFR) dilution attributable to cell proliferation was assessed by gating the lymphocyte population using flow cytometry.

### Cytokine production

αB7-H3-αCD3 ENG T cells and un-transduced T cells (UTD T) were co-cultured with target cells at a 5:1 E:T ratio in a serum-free medium for 24 h. Subsequent to co-cultivation, culture supernatants were harvested, subjected to centrifugation to remove cell debris, and preserved at − 20 °C. The concentrations of cytokines within the culture supernatants were assessed utilizing the LEGENDplex™ Human CD8/NK cell panel (#741065, BioLegend), facilitating the simultaneous quantification of 13 human cytokines and proteins, comprising IL-2, IL-4, IL-6, IL-10, IL-17A, IFN- γ, TNF- α, soluble Fas, soluble FasL, granzyme A, granzyme B, perforin, and granulysin, through the Cytokine Bead Array (CBA) methodology, following the manufacturer's instructions. The samples were analyzed using a CytoFLEX flow cytometer (Becton Dickinson (BD) Biosciences, New Jersey, USA).

### Cytotoxic activity of bystander T cells

The target cells were labeled with 0.75 µM of CellTracker™ Orange CMRA Dye (Thermo Fisher Scientific). Co-culture assays were conducted by incubating PHA-L activated T cells with CMRA-labeled target cells at E:T ratios of 1:1, 5:1, and 10:1 and adding culture supernatant from either parental HEK293T cells or HEK293T cells stably expressing αB7-H3-αCD3 BITEs with varying volume for 24 h. Following co-cultivation, counting beads (123count™ eBeads Counting Beads; Thermo Fisher Scientific) were introduced to each sample to facilitate the quantification of the absolute number of target cells via flow cytometry analysis, following the manufacturer's protocol. The percentage of tumor cell cytotoxicity was calculated using the formula: (1 − (target cells in each condition/target cells alone at the indicated time)) × 100.

### Statistical analysis

The effects of various factors on the measured variables were thoroughly analyzed using Student's t-tests, one-way ANOVA, and two-way repeated-measures ANOVA. To identify pairwise differences between group means, Tukey's post hoc tests were applied. Each experiment was performed in triplicate (N = 3) or with additional groups to ensure data reliability and reproducibility. Statistical significance was defined as *p* < 0.05 for all analyses. Results are reported as means ± standard error of the mean (SEM). All statistical analyses were conducted using GraphPad Prism 10.0 (GraphPad Software, San Diego, CA, USA).

## Results

### Cell surface expression of B7-H3 of target cell lines

**Fig. 1:**
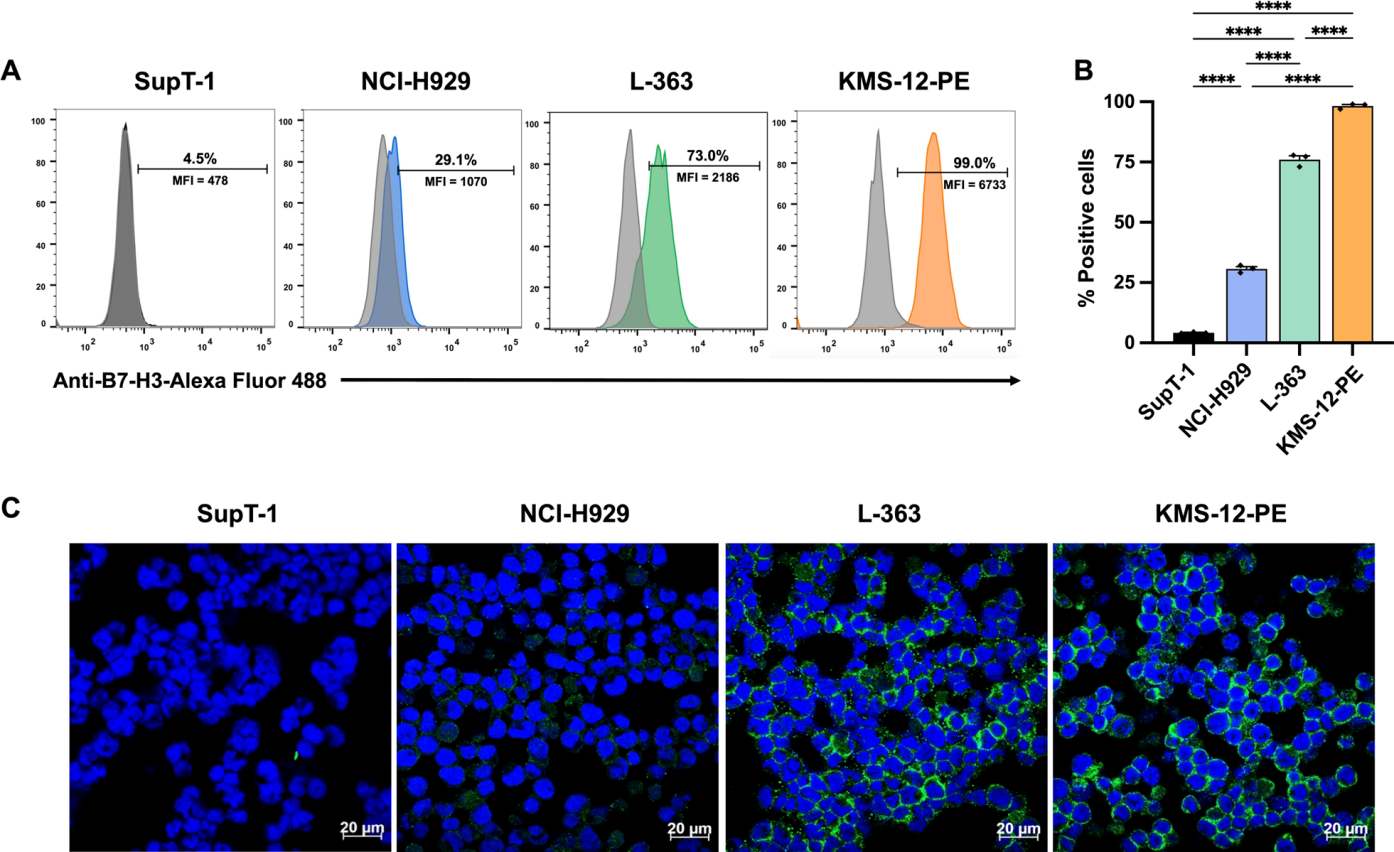
B7-H3 expression in cell lines. The expression levels of B7-H3 were evaluated through flow cytometry and immunofluorescence assay (IFA) across a spectrum of cell lines, encompassing a non-multiple myeloma (non-MM) cell line (SupT-1) and multiple myeloma (MM) cell lines (NCI-H929, L-363, and KMS-12-PE). **A** Flow cytometry histogram illustrating surface B7-H3 expression relative to an isotype control (light gray). **B** Quantification of the percentage of cells positive for B7-H3 expression. **C** Immunofluorescence assay (IFA) depicted surface expression of B7-H3 in SupT-1, NCI-H929, L-363, and KMS-12-PE cell lines. Anti-B7-H3 staining, followed by Alexa fluor 488-conjugated secondary antibody, is represented in green, while nuclear staining using Hoechst 33342 is denoted in blue. The data were derived from three independent experiments (N = 3) and are presented as the mean, ± standard error of the mean (SEM). Statistical significance was determined using one-way analysis of variance (ANOVA) with Tukey's post-hoc test (*p < 0.05, **p < 0.01, ***p < 0.001, ****p < 0.0001)

The cell surface expression of B7-H3 was assessed in various target cell lines, namely multiple myeloma (MM) cell lines NCI-H929, L-363, and KMS12-PE, utilizing flow cytometry and immunofluorescence assay (IFA) with the specific B7-H3 antibody (F-11). The B7-H3 negative expression cell line, SupT-1, derived from lymphoblastic lymphoma (T lymphoblast), served as a comparative control. Flow cytometric analysis revealed distinct levels of B7-H3 surface expression. Notably, SupT-1 exhibited negligible surface B7-H3 expression (4.2 ± 0.2%), contrasting starkly with the MM cell lines NCI-H929, L-363, and KMS-12-PE, which manifested surface B7-H3 expressions of 30.7 ± 0.9% (*p* < 0.0001), 76.0 ± 1.5% (*p* < 0.0001), and 98.3 ± 0.6% (*p* < 0.0001), respectively (Fig. 1A, B). Additionally, the surface expression of B7-H3 in MM cells was corroborated using immunofluorescence assay (IFA), where green fluorescence denoted B7-H3 on the cell surface, and blue fluorescence represented the cell nuclei (Fig. 1C). These findings concurred with flow cytometry results, delineating SupT-1 as B7-H3 negative, NCI-H929 as exhibiting low B7-H3 expression, L-363 as manifesting medium to high B7-H3 expression, and KMS-12-PE as displaying high B7-H3 expression on the cell surface (Fig. 1C).

### Construction lentiviral vector expressing αB7-H3-αCD3 BITE

**Fig. 2:**
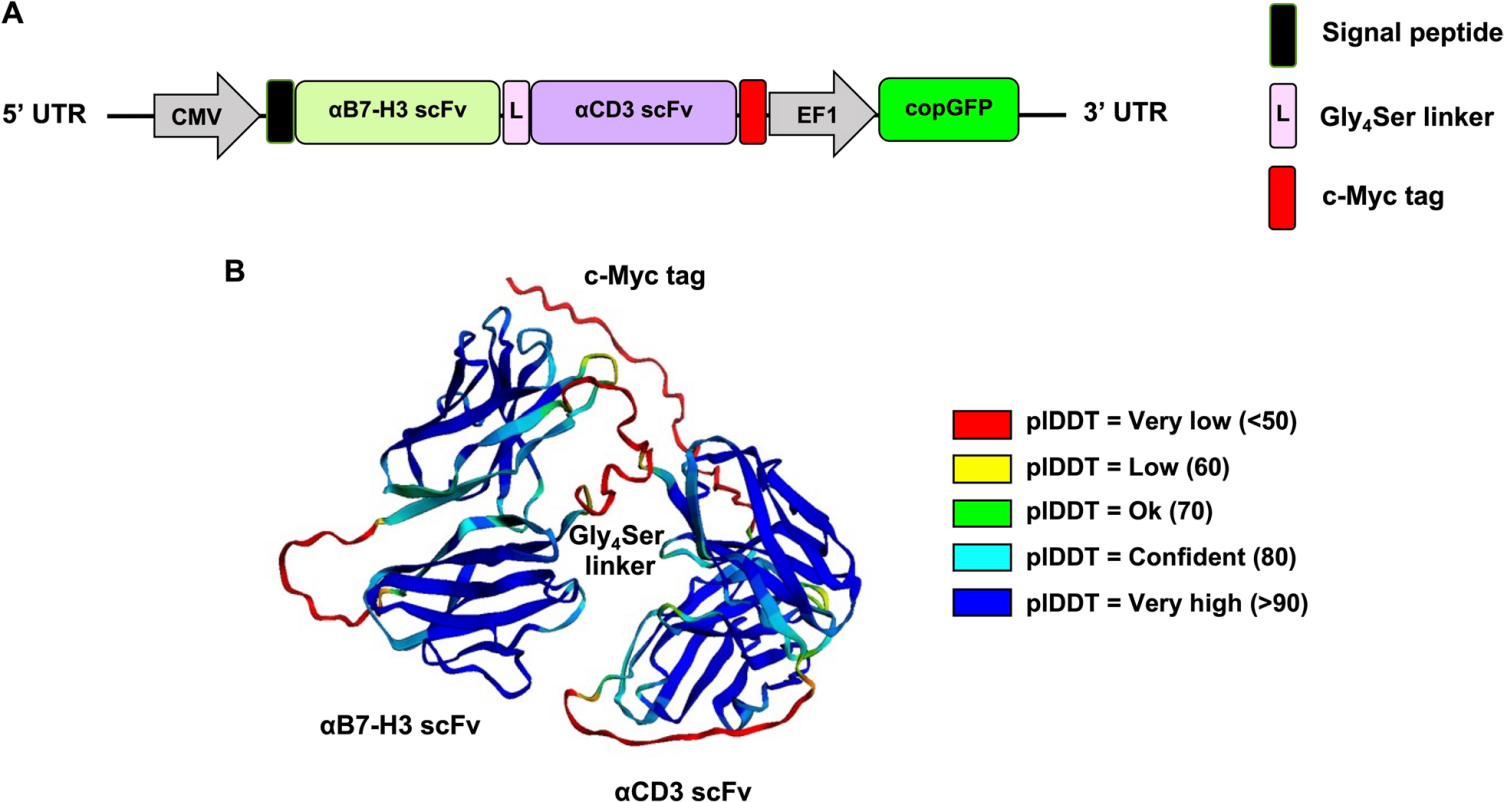
Constructs of αB7-H3-αCD3 BITE. **A** Illustration depicting the lentiviral plasmid construct encoding αB7-H3-αCD3 BITE, including the incorporation of a myc-tag and copGFP fluorescent protein. (B) AlphaFold prediction and resultant protein structure visualization for αB7-H3-αCD3 BITE

To generate an αB7-H3-αCD3 bispecific T cell engager (αB7-H3-αCD3 BITE) targeting the B7-H3 antigen on tumor cells and CD3 on T cells, we employed restriction endonuclease cleavage and cloning methodologies to construct a lentiviral plasmid harboring the cDNA encoding the αB7-H3-αCD3 BITE protein. Figure 2A illustrates the schematic representation of the αB7-H3-αCD3 BITE lentiviral transfer plasmid. The αB7-H3-αCD3 BITE was engineered under the control of the cytomegalovirus (CMV) promoter, incorporating the humanized αB7-H3 single-chain variable fragment (scFv) derived from Ahmed, M. et al. (hu8H9) [31], linked to the αCD3 scFv sequence (obtained from the OKT3 αCD3 monoclonal antibody) via G4S linkers (L). This construct was then fused in frame with the Myc tag sequence, followed by the inclusion of a copGFP reporter driven by the EF1 promoter at the 3’ terminus. Additionally, we utilized AlphaFold for protein structure prediction from the respective protein sequences (Fig. 2B). The AlphaFold model provides confidence estimations in the form of predicted local Distance Difference Test (lDDT) scores per residue [33]. The lDDT score is a superposition-free metric ranging from 0 to 100, indicating the fidelity of the protein model to the reference structure [34]. The overall confidence for the entire protein chain (plDDT) is determined by averaging all residues-specific lDDT scores. A graphical representation of the αB7-H3-αCD3 BITE constructs, segmented according to αB7-H3 scFv and αCD3 scFv and individually superimposed onto the target structure, is presented. The color scheme of the model corresponds to plDDT scores, with sky blue denoting confident values and navy blue representing exceedingly high values of lDDT.

### αB7-H3-αCD3 BITE expression, secretion, and binding proficiency

**Fig. 3:**
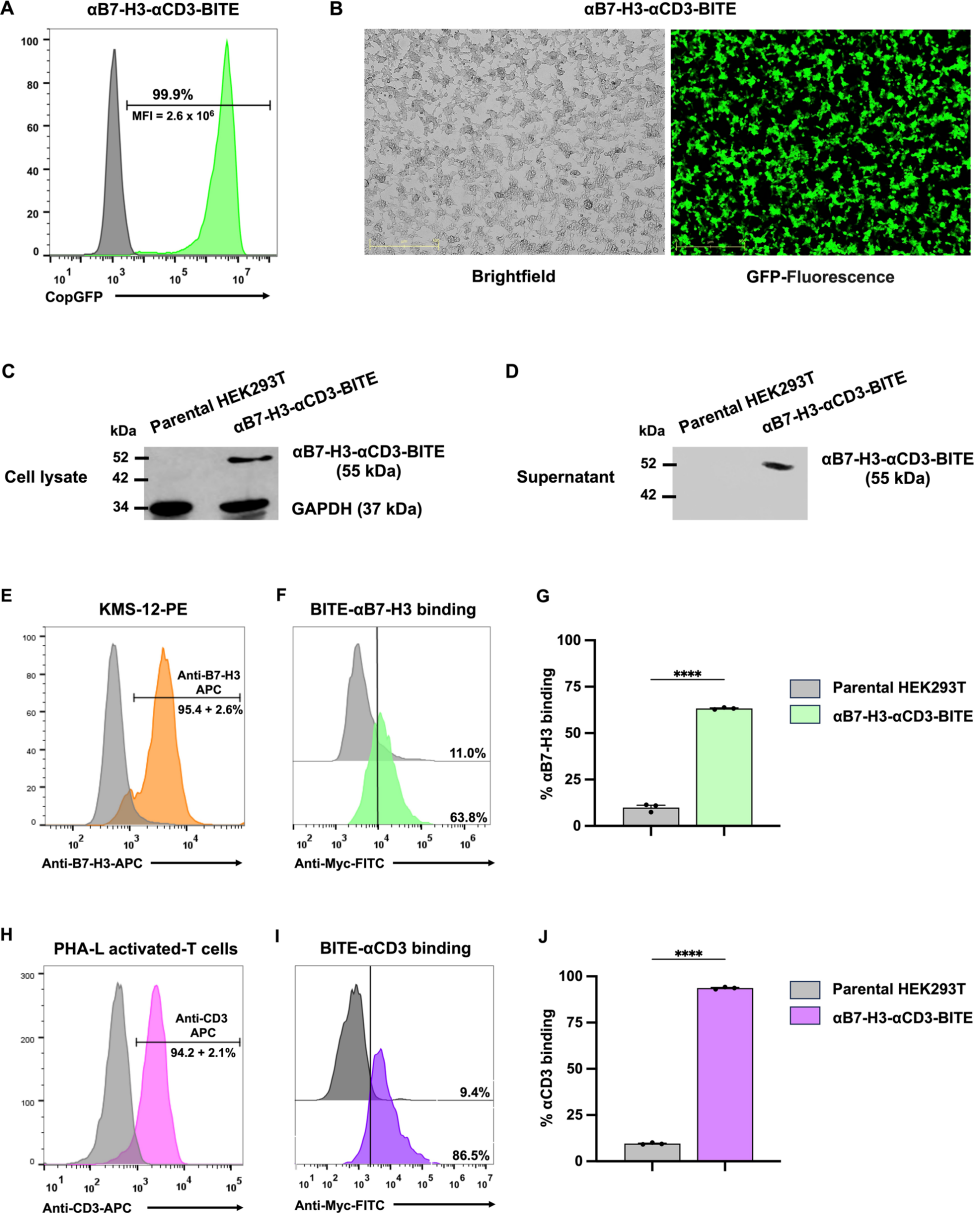
Expression, secretion, and binding proficiency of αB7-H3-αCD3 BITE. **A** Representative histogram depicting copGFP fluorescent expression in HEK293T cells stably expressing αB7-H3-αCD3 BITE, analyzed via flow cytometry. **B** Visualization of copGFP expression in HEK293T cells stably expressing αB7-H3-αCD3 BITE using bright field and GFP-fluorescence images obtained with muvicyte. **C** Immunoblotting analysis with an anti-c-Myc tag antibody demonstrating αB7-H3-αCD3 BITE protein presence in cell lysates and **D** culture supernatants of HEK293T cells stably expressing αB7-H3-αCD3 BITE. **E** Histogram illustrating B7-H3 positivity in KMS-12-PE cell line. **F** Histogram depicting the direct binding proficiency of αB7-H3-αCD3 BITE to B7-H3 on KMS-12-PE cells. **G** Bar graph summarizing the percentage of positive cells detected by anti-Myc FITC. **H** Histogram showing CD3 positivity in PHA-L activated T cells. **I** Histogram depicting the direct binding capacity of αB7-H3-αCD3 BITE to CD3 on PHA-L-activated T cells. **J** Bar graph representing the percentage of positive cells detected by anti-Myc FITC. The data were collected from at least three independent experiments (N = 3) and presented as mean, ± standard error of the mean (SEM). Statistical significance was determined using unpaired Student's t-tests (*p < 0.05, **p < 0.01, ***p < 0.001, **** *p* < 0.0001).

To assess the protein expression of αB7-H3-αCD3 BITE, the αB7-H3-αCD3 BITE plasmid was utilized to produce lentivirus in HEK293T cells. Subsequently, the lentivirus harboring αB7-H3-αCD3 BITE was transduced into HEK293T cells to establish stable cell lines expressing αB7-H3-αCD3 BITE. Initial examination via flow cytometry revealed CopGFP expression in transduced cells (Fig. 3A), and subsequent analysis involved capturing brightfield and fluorescence images using MuviCyte (Fig. 3B). The findings demonstrated a copGFP green fluorescence expression of 99.9% with a mean fluorescence intensity (MFI) of 2.6 × 10^6^ in HEK293T cells stably expressing αB7-H3-αCD3 BITE (Fig. 3A). Moreover, these stably expressing cells exhibited copGFP green fluorescence uniformly across all areas compared to brightfield images (Fig. 3B). Immunoblotting analysis employing an anti-c-Myc antibody confirmed the expression of αB7-H3-αCD3 BITE protein at 55 kDa in both cell lysate and culture supernatant of HEK293T cells stably expressing αB7-H3-αCD3 BITE (Fig. 3C and D), with a concentration of 45.8 µg/mL detected in the supernatant (Additional file 1). This observation indicates successful translation and secretion of αB7-H3-αCD3 BITE into the culture supernatant. Further characterization of the secreted αB7-H3-αCD3 BITE involved assessing its binding affinity towards target molecules. The binding ability of αB7-H3-αCD3 BITE was evaluated using B7-H3-expressing KMS-12-PE cells (Fig. 3E), and CD3^+^ PHA-L activated T cells (Fig. 3H). Following incubation with αB7-H3-αCD3 BITE, both KMS-12-PE cells and CD3^+^ PHA-L activated T cells exhibited positive cell surface presence of αB7-H3-αCD3 BITE, as evidenced by anti-c-Myc tag antibody staining and flow cytometry analysis (Fig. 3F, G, I, J). The results indicated specific binding of αB7-H3-αCD3 BITE to B7-H3 and CD3, with binding percentages of 63.3 ± 0.3% and 93.7 ± 0.3%, respectively, compared to parental HEK293T cells (10.0 ± 1.3% and 9.6 ± 0.3%), all with statistical significance (*p* < 0.0001). These findings suggest the specificity of αB7-H3-αCD3 BITE in binding to both B7-H3 on KMS-12-PE cells and CD3 on T cells.

### Development and characterization of αB7-H3-αCD3 ENG T cells

**Fig. 4:**
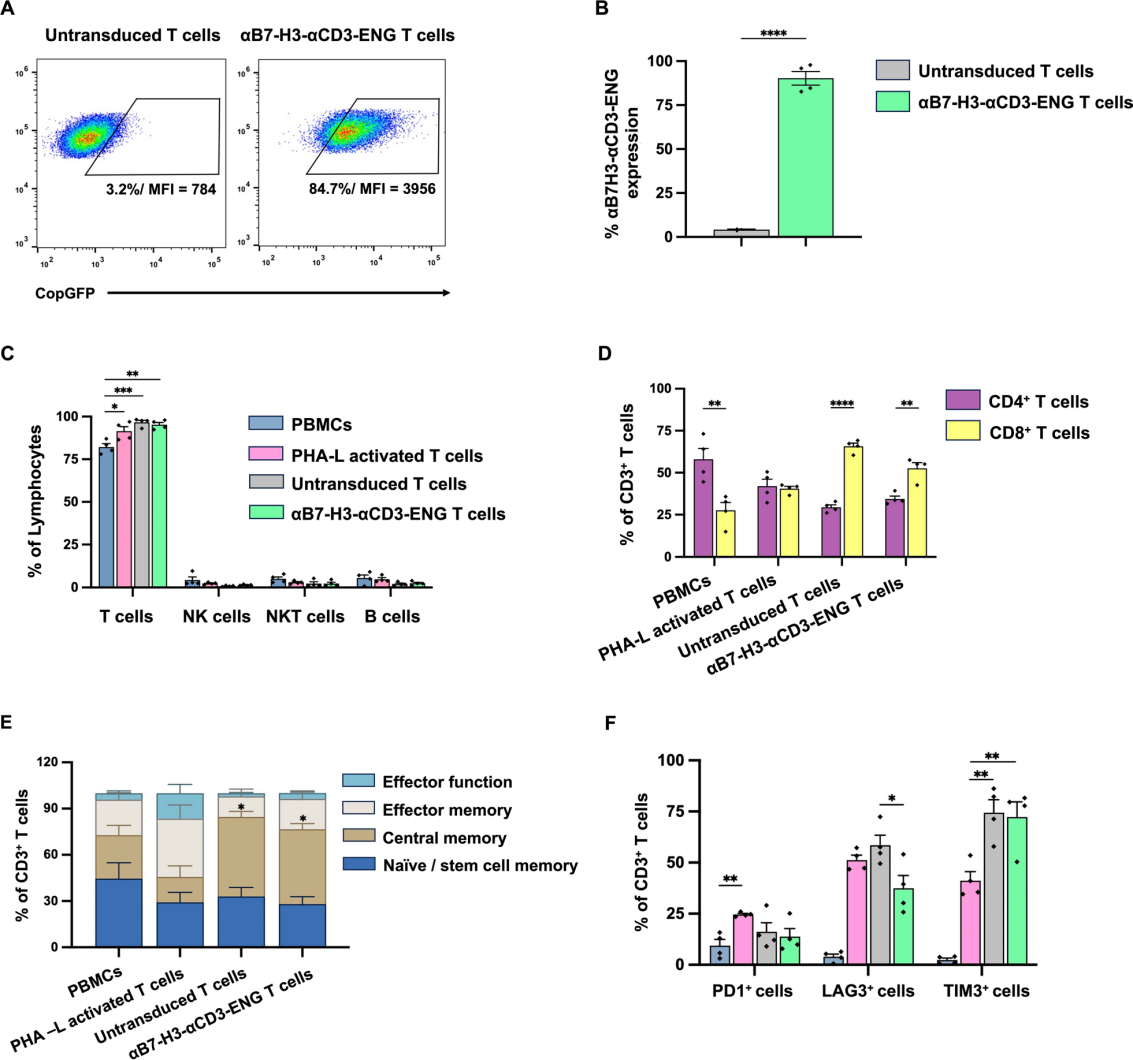
Generation and characterization of αB7-H3-αCD3 ENG T cells. **A** Histogram plots demonstrate the transduction efficiency of αB7-H3-αCD3 ENG constructs, expressing anti- αB7-H3-αCD3 ENG protein on T cells isolated from PBMCs of healthy donors, detected by copGFP fluorescence. **B** Summary data from 4 different healthy donors illustrating transduction efficiency. **C** Immune cell populations are distinguished by CD3^+^ (T cells), CD3^Neg^CD56^+^CD16^+/Neg^ (NK cells), CD3^+^CD56^+^ (NKT cells), and CD3^Neg^CD19^+^ (B cells). **D** Percentages of helper (CD3^+^CD4^+^) T cells and cytotoxic (CD3^+^CD8^+^) T cells. **E** Flow cytometric analysis of T cells subset on CD3^+^ lymphocytes. **F** Evaluation of three exhaustion markers, PD-1, LAG-3, and TIM-3, in CD3^+^ cells using flow cytometry. The data were obtained from 4 independent experiments (N = 4), and the results are presented as mean, ± standard error of the mean (SEM). Statistical analyses were performed using unpaired Students' t-tests for comparison between two groups, one-way ANOVA with Tukey's post-hoc test for comparison between three or more groups, and 2-way repeated-measures ANOVA for the analysis of T cell subsets (*p < 0.05, **p < 0.01, ***p < 0.001, and ****p < 0.0001)

The present study outlines the production of T cells engineered to secrete αB7-H3-αCD3 BITE through lentiviral transduction of primary human T cells using lentiviral particles carrying genes encoding the respective BITEs. Transduction efficiencies were evaluated by quantifying copGFP expression in T cells, revealing that 4.2 ± 0.4% of untransduced T cells (UTD T) expressed αB7-H3-αCD3 BITE, while 90.1 ± 3.8% of T cells expressed the construct (Fig. 4A, B). Immunophenotypic analysis of αB7-H3-αCD3 ENG T cells showed that over 90% of the T cell population expressed CD3 (Fig. 4C). Conversely, the NK, NKT, and B cell populations were minimal and exhibited no significant differences among the experimental groups. Subsequent examination of the CD3^+^ population revealed a higher prevalence of cytotoxic T cells (CD8^+^) compared to helper T cells (CD4^+^) in both UTD T cells (65.7 ± 1.9% and 29.4 ± 1.5%, respectively) and αB7-H3-αCD3 ENG T cells (52.6 ± 3.4% and 34.4 ± 1.7%, respectively) (Fig. 4D). Analysis of T cell subsets, including naive/stem cell memory, central memory, effector memory, and effector function cells, demonstrated significantly higher central memory proportions in both UTD T cells (51.7 ± 3.5%) and αB7-H3-αCD3 ENG T cells (48.7 ± 3.6%) compared to PHA-L activated-T cells (16.5 ± 7.1%), with a *p*-value of 0.0314 and 0.0425, respectively (Fig. 4E). Additionally, analysis of the exhaustion marker LAG-3 expression revealed significantly lower levels in αB7-H3-αCD3 ENG T cells (37.4 ± 6.3%) compared to UTD T cells (58.5 ± 4.9%, *p* = 0.0185). However, the expression levels of PD-1 and TIM-3 were not substantially different from those observed in UTD T cells (Fig. 4F).

### Anti-tumor activities of αB7-H3-αCD3 ENG T cells against multiple myeloma cells expressing B7-H3

**Fig. 5:**
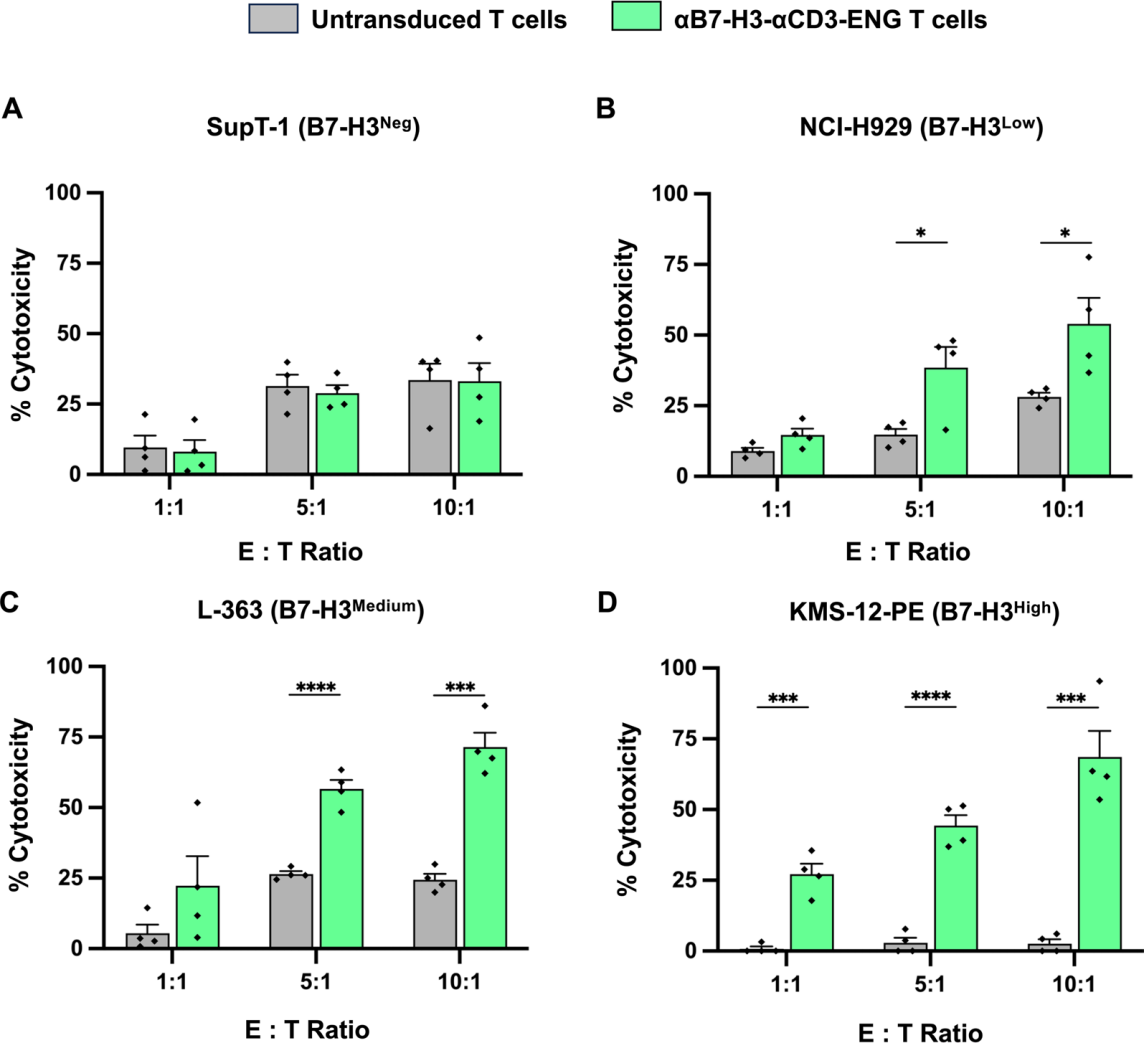
Anti-tumor activity of αB7-H3-αCD3 ENG T cells against B7-H3-expressing multiple myeloma. The killing activities of both untransduced (UTD) T cells and αB7-H3-αCD3 ENG T cells were evaluated against **A** SupT-1 (B7-H3^neg^), **B** NCI-H929 (B7-H3^low^), **C** L-363 (B7-H3^medium^), and **D** KMS-12-PE (B7-H3^high^) cells. Co-cultures were maintained at effector-to-target (E:T) ratios of 1:1, 5:1, and 10:1 for 24 h, followed by counting the remaining target cells using counting beads and analysis by flow cytometry. Data were collected from 4 independent experiments (N = 4), and results are presented as mean, ± standard error of the mean (SEM). Statistical significance was determined using unpaired Student's t-tests (*p < 0.05, **p < 0.01, ***p < 0.001, and ****p < 0.0001)

This experiment aimed to assess the anti-tumor activities of T cells engineered to secrete αB7-H3-αCD3 BITEs against multiple myeloma (MM) cell lines expressing B7-H3. SupT-1 cells served as negative control target cells (B7-H3^neg^), while NCI-H929 (B7-H3^low^), L-363 (B7-H3^medium^), and KMS-12-PE (B7-H3^high^) cells were selected as MM target cells expressing varying levels of B7-H3. Target cells were co-cultured with either untransduced (UTD) T cells or αB7-H3-αCD3 ENG T cells at different effector-to-target (E:T) ratios (1:1, 5:1, and 10:1) for 24 h, after which the viability of target cells was assessed using flow cytometry. Results demonstrated that αB7-H3-αCD3 ENG T cells exhibited minimal cytotoxicity against SupT-1 (B7-H3^neg^) cells compared to control UTD T cells (Fig. 5A). However, αB7-H3-αCD3 ENG T cells demonstrated dose-dependent killing of B7-H3-expressing MM cells, including NCI-H929, L-363, and KMS-12-PE (Fig. 5B–D). The cytotoxicity of αB7-H3-αCD3 ENG T cells against NCI-H929 cells reached up to 38.5 ± 7.4% (*p* = 0.0212) and 54.0 ± 9.2% (*p* = 0.0317) at an E:T ratio of 5:1 and 10:1, respectively, compared to control UTD T cells (14.7 ± 2.1% and 28.1 ± 1.5%), respectively (Fig. 5B). Similarly, the percentage of cytotoxicity against L-363 cells by αB7-H3-αCD3 ENG T cells was observed to be up to 56.6 ± 3.2, (*p* < 0.0001) and 71.4 ± 5.2% (*p* = 0.0002) at an E:T ratio of 5:1 and 10:1, respectively, compared to control UTD T cells (26.5 ± 1.0% and 24.4 ± 2.1%), respectively (Fig. 5C). Notably, αB7-H3-αCD3 ENG T cells exhibited significant cytotoxic effects against high B7-H3-expressing KMS-12-PE cells starting from the E:T ratio of 1:1, with cytotoxicity reaching 27.2 ± 3.7% (*p* = 0.0004), 44.4 ± 3.7% (*p* < 0.0001) and 68.6 ± 9.2% (*p* = 0.0004) at E:T ratios of 1:1, 5:1, and 10:1, respectively, compared to control UTD T cells (0.8 ± 0.8%, 2.9 ± 1.9% and 2.6 ± 1.5%), respectively (Fig. 5D).

### Stimulation of αB7-H3-αCD3 ENG T cell proliferation via co-culture with B7-H3-expressing multiple myeloma cells

**Fig. 6:**
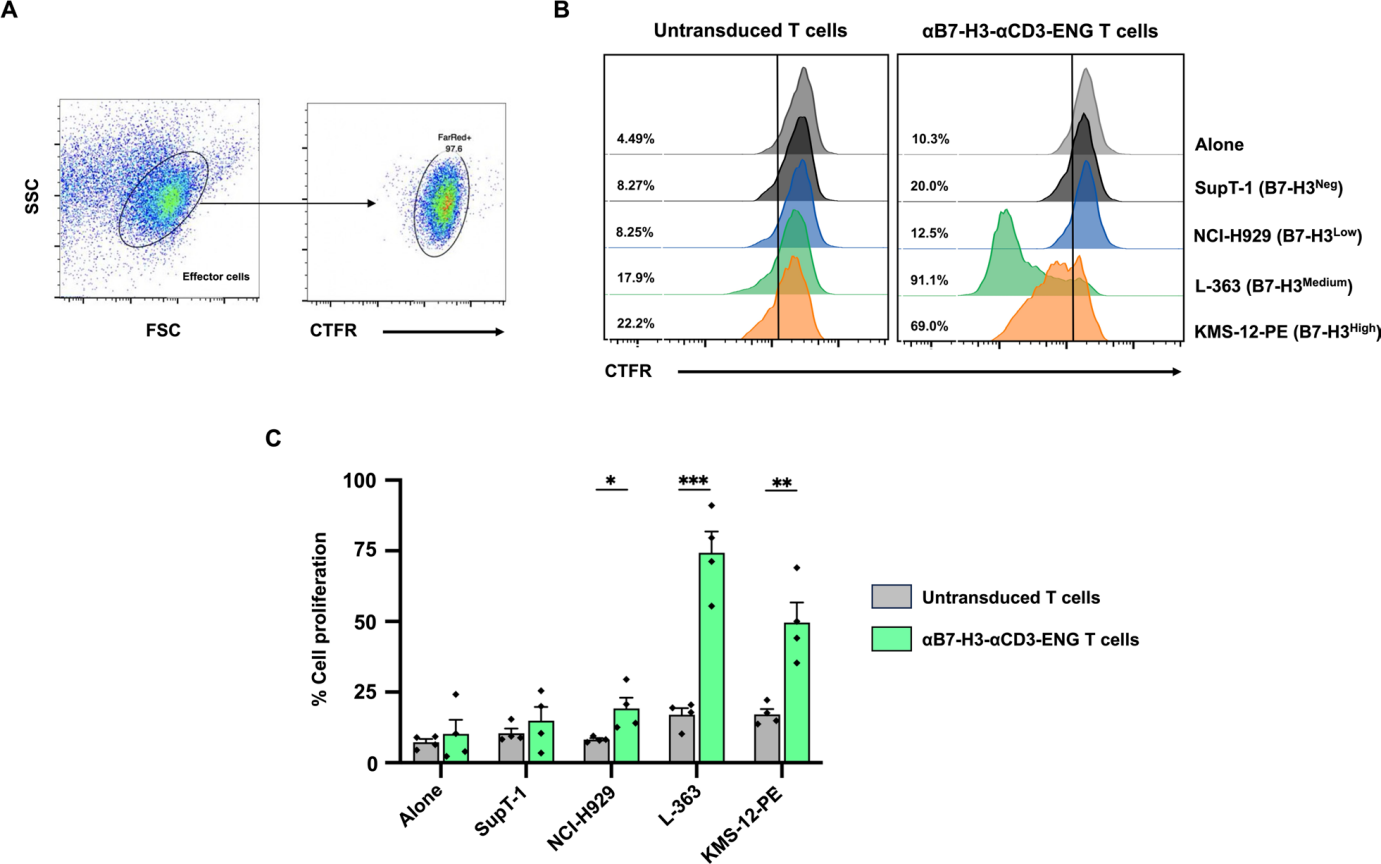
Proliferation activity of αB7-H3-αCD3 ENG T cells against B7-H3-expressing multiple myeloma. **A** Gating strategy for the quantitation of CellTrace™Far Red (CTFR) dilution **B** Histogram illustrating the proliferation activity and **C** percentage of cell proliferation of both untransduced (UTD) T cells and αB7-H3-αCD3 ENG T cells. The cells were activated by co-culturing without target cells (Alone) or with target cells: SupT-1 (B7-H3^neg^), NCI-H929 (B7-H3^low^), L-363 (B7-H3^medium^), and KMS-12-PE (B7-H3^high^) at an effector to target (E:T) ratio of 5:1 for five days in the absence of exogenous cytokines. Cell proliferation was assessed by CellTrace™Far Red (CTFR) dilution using flow cytometry. Data were collected from 4 independent experiments (N = 4), and results are presented as mean, ± standard error of the mean (SEM). Statistical significance was determined using unpaired Student's t-tests (*p < 0.05, **p < 0.01, ***p < 0.001, and ****p < 0.0001)

To assess cell proliferation, αB7-H3-αCD3 ENG T cells were labeled with CellTrace™ Far Red (CTFR) dye before co-culturing with SupT-1 (B7-H3^neg^), NCI-H929 (B7-H3^low^), L-363 (B7-H3^medium^), and KMS-12-PE (B7-H3^high^) cells at an effector to target (E:T) ratio of 5:1. After five days, the reduction in CTFR signal within αB7-H3-αCD3 ENG T cells was quantified using flow cytometry, depicting the histogram of sequential CTFR stain halving (Fig. 6A, B) and the percentage of CTFR dilution (Fig. 6C). Experimental results indicated that αB7-H3-αCD3 ENG T cells did not exhibit significant proliferation compared to untransduced (UTD) T cells when cultured alone or with negative (SupT-1) cells (Fig. 6C). However, co-culture with target cells expressing varying levels of the B7-H3 antigen (NCI-H929, L-363, and KMS-12-PE) resulted in significantly enhanced proliferation of αB7-H3-αCD3 ENG T cells compared to UTD T cells. Specifically, the proliferation rates of αB7-H3-αCD3 ENG T cells against the NCI-H929, L-363, and KMS-12-PE cells were 19.2 ± 3.9% (*p* = 0.0309), 74.4 ± 7.5% (*p* = 0.0003), and 49.6 ± 7.1% % (*p* = 0.0045), respectively, compared to UTD T cells (8.2 ± 0.5%, 17.0 ± 2.3%, and 17.1 ± 1.9%, respectively) (Fig. 6C).

### Cytokine production of αB7-H3-αCD3 ENG T cells in response to B7-H3 expressing multiple myeloma cells

**Fig. 7:**
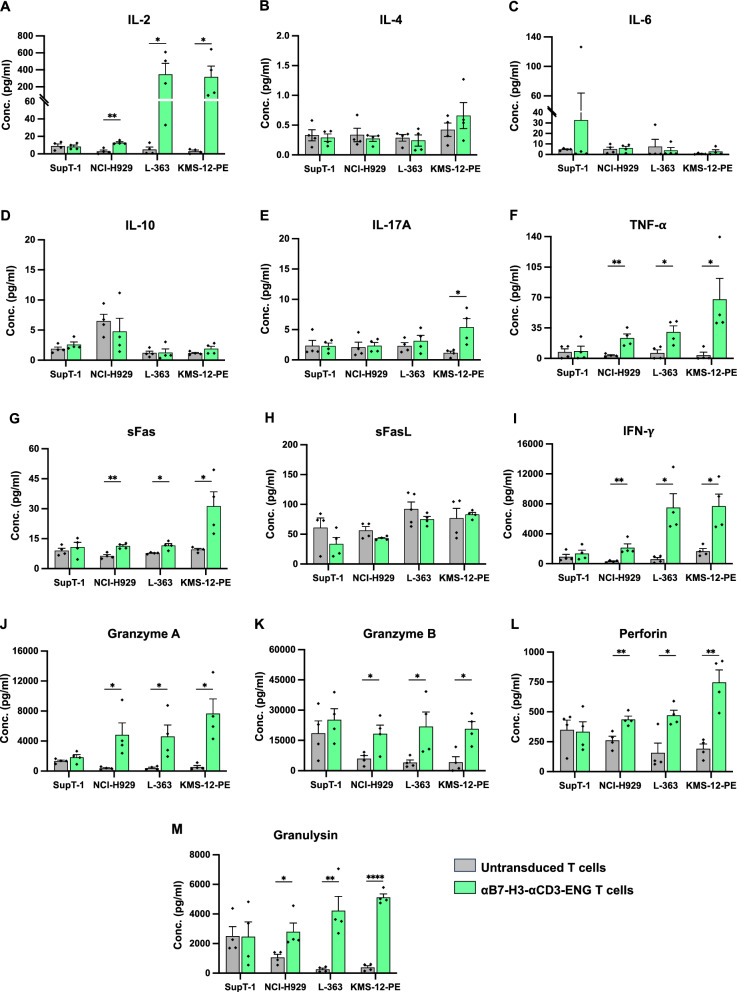
Cytokine production by αB7-H3-αCD3 ENG T cells against B7-H3-expressing Multiple myeloma (MM) cell lines. **A** IL-2, **B** IL-4, **C** IL-6, **D** IL-10, **E** IL-17A, **F** TNF-α, **G** sFas, **H** sFasL **I** IFN-γ, **J** Granzyme-A, **K** Granzyme-B, **L** Perforin, and **M** Granulysin production levels in the cell culture supernatants of αB7-H3-αCD3 ENG T cells. These cells were activated by culturing with SupT-1 (B7-H3^neg^), NCI-H929 (B7-H3^low^), L-363 (B7-H3^medium^), and KMS-12-PE (B7-H3^high^) cells at an effector-to-target (E:T) ratio of 5:1 for 24 h, and cytokine levels were analyzed by cytometric bead array (CBA). Data were collected from 4 independent experiments (N = 4), and results are presented as mean ± standard error of the mean (SEM). Statistical significance was determined using unpaired Student's t-tests (*p < 0.05, **p < 0.01, ***p < 0.001, and ****p < 0.0001)

This study explored cytokine production in response to B7-H3-expressing multiple myeloma (MM) cells using SupT-1 (B7-H3^neg^), NCI-H929 (B7-H3^low^), L-363 (B7-H3^medium^), and KMS-12-PE (B7-H3^high^) cells co-cultured with untransduced (UTD) T cells or αB7-H3-αCD3 ENG T cells at an effector-to-target (E:T) ratio of 5:1. Cytokine levels were quantified using LEGENDplex™ Human CD8/NK cell panel Cytokine Bead Array (CBA), assessing 13 cytokines and proteins after 24 h of co-culture. The online resource presents the cytokine concentrations of αB7-H3-αCD3 ENG T cells compared to UTD T cells when co-cultured with SupT-1, NCI-H929, L-363, and KMS-12-PE cells. Results indicated that cytokine concentrations of αB7-H3-αCD3 ENG T cells against B7-H3-negative SupT-1 cells were not significantly elevated compared to UTD T cells (Fig. 7). However, in co-cultures with B7-H3-expressing cells (NCI-H929, L-363, and KMS-12-PE), levels of IL-2, TNF-α, sFas, IFN-γ, granzyme A, granzyme B, perforin, and granulysin in the culture media were significantly increased in αB7-H3-αCD3 ENG T cells compared to UTD T cells. (Fig. 7). Notably, IL-17A levels exhibited a remarkable increase in the culture media of αB7-H3-αCD3 ENG T cells co-cultured with B7-H3 expression as KMS-12-PE cells, which displayed high B7-H3 expression, compared to the UTD T cells (Fig. 7). Detailed cytokine concentration levels are provided in Additional file 2.

### Cytotoxic activity of bystander T cells against B7-H3-expressing multiple myeloma (MM) cell lines

**Fig. 8:**
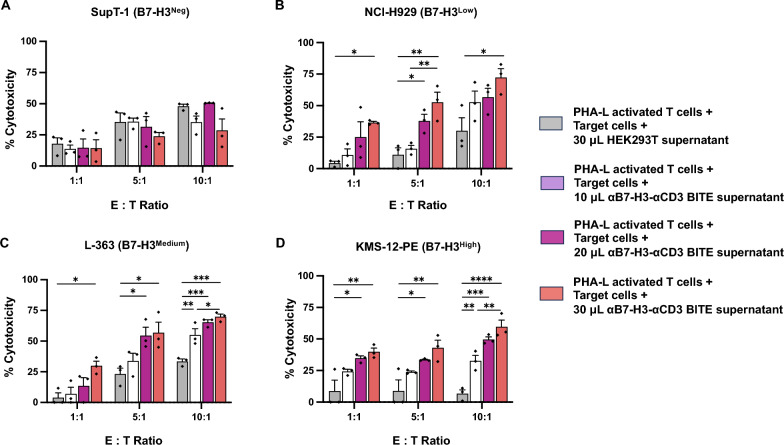
Cytotoxic activity of bystander T cells against B7-H3-expressing multiple myeloma (MM) cell lines. Bystander T cells derived from PHA-L activated T cells were combined with varying volumes of culture supernatant of the HEK293T cells stably expressing αB7-H3-αCD3 BITEs to assess cytotoxicity against **A** SupT-1 (B7-H3^neg^), **B** NCI-H929 (B7-H3^low^), **C** L-363 (B7-H3^medium^), and **D** KMS-12-PE (B7-H3^high^) cells. Co-cultures were maintained at effector-to-target (E:T) ratios of 1:1, 5:1, and 10:1 for 24 h, followed by quantification of remaining target cells using counting beads and analysis by flow cytometry. Data represent the mean ± standard error of the mean (SEM) from 4 independent experiments (N = 4). Statistical significance was determined using one-way ANOVA with Tukey's post-hoc test (*p < 0.05, **p < 0.01, ***p < 0.001, and ****p < 0.0001)

To demonstrate the cytotoxic activity of bystander T cells redirected by αB7-H3-αCD3 BITEs towards B7-H3-positive target cells, we conducted experiments using culture supernatants of HEK293T cell stably expressing αB7-H3-αCD3 BITEs. These culture supernatants, with varying volumes, were mixed with PHA-L activated T cells and B7-H3-positive tumor cells at three effector-to-target (E:T) ratios. After 24 h, the absolute number of target cells was quantified using counting beads. Results indicated that PHA-L-activated T cells with αB7-H3-αCD3 BITEs exhibited minimal cytotoxicity against SupT-1 (B7-H3^neg^) cells compared to the negative controls (Fig. 8A). However, these T cells demonstrated effective killing of B7-H3-expressing MM cells, including NCI-H929, L-363, and KMS-12-PE, in a dose-dependent manner (Fig. 8B–D). For instance, the cytotoxicity of PHA-L-activated T cells with 30 µL of αB7-H3-αCD3 BITE supernatant against NCI-H929 (B7-H3^low^) cells was significantly increased at all E:T ratios (1:1, 5:1, and 10:1) compared to negative controls, with percentages of 36.5 ± 1.0% (*p* = 0.0357), 52.6 ± 8.1% (*p* = 0.0039), and 72.3 ± 7.1% (*p* = 0.0306), compared to the negative control (4.3 ± 1.6%, 11.0 ± 5.6%, 30.1 ± 10.2%, respectively) (Fig. 8B). Similarly, the cytotoxicity against L-363 (B7-H3^medium^) cells with 30 μL of supernatant was notable at E:T ratios of 1:1, 5:1, and 10:1, with percentages of 29.8 ± 3.8% (*p* = 0.0297), 56.8 ± 8.6% (*p* = 0.0318) and 69.8 ± 2.2% (*p* = 0.0002), respectively (Fig. 8C). Notably, PHA-L-activated T cells demonstrated significant cytotoxic effects against KMS-12-PE (B7-H3^High^) cells even at an E:T ratio of 1:1, with percentages of 39.9 ± 3.0% (*p* = 0.0075), 43.1 ± 6.0% (*p* = 0.0083), and 59.7 ± 5.4% (*p* < 0.0001) at E:T ratios of 1:1, 5:1, and 10:1, respectively (Fig. 8D). Interestingly, reducing the volume of the αB7-H3-αCD3 BITE supernatant to 20 μL significantly increased target cell destruction compared to the control group. For instance, at the E:T of 1:1, PHA-L-activated T cells with αB7-H3-αCD3 BITE supernatant showed effective killing of high B7-H3-expressing MM cells (KMS-12-PE) with a percentage of 34.7 ± 1.9% (*p* = 0.0205; Fig. 8D). When increasing the effector cells to 5:1, these T cells effectively killed low (NCI-H929; Fig. 8B) and medium (L-363; Fig. 8C) B7-H3-expressing MM cells, with percentages of 37.7 ± 5.4% (*p* = 0.0436), 54.5 ± 6.7% (*p* = 0.0443), respectively. Furthermore, adding 10 µL of αB7-H3-αCD3 BITE supernatant into PHA-L-activated T cells at E:T ratio of 10:1 resulted in highly significant cytotoxicity against L-363 (B7-H3^medium^; Fig. 8C) and KMS-12-PE (B7-H3^high^; Fig. 8D) cells, with percentages of 55.0 ± 5.2% (*p* = 0.0056), 32.7 ± 4.4%, and (*p* = 0.0067) compared to the negative controls (33.4 ± 2.0%, and 6.7 ± 3.0%, respectively). Additionally, increasing the volume of αB7-H3-αCD3 BITE supernatant to 30 μL at an E:T ratio of 10:1 resulted in highly significant cytotoxicity against L-363 (B7-H3^medium^; Fig. 8C) and KMS-12-PE (B7-H3^high^; Fig. 8D) cells, with percentages of 69.8 ± 2.2% (*p* = 0.0417) and 59.7 ± 5.4%, (*p* = 0.0054) compared to 10 μL of supernatant and PHA-L-activated T cells at an E:T ratio of 10:1 (55.0 ± 5.2%, and 32.7 ± 4.4%), respectively.

## Discussion

Multiple myeloma (MM) is a severe hematologic cancer with high mortality. Despite advances in treatments, drug resistance remains a significant challenge, particularly in relapsed/refractory MM (RRMM). Immunotherapies like monoclonal antibodies and CAR-T cell therapies show promise but face issues such as cytokine release syndrome and neurotoxicity. Bispecific T-cell engagers (BITEs) are effective but require continuous infusion due to their short half-life. BITE therapies targeting BCMA/CD3, such as AMG 701, combined with immunomodulatory drugs (IMiDs) like lenalidomide and pomalidomide, have demonstrated enhanced anti-MM cytotoxicity and immunomodulatory effects in preclinical models [35]. However, targeting BCMA with BITE therapies can face challenges due to BCMA loss, mutations, or reduced BCMA density on plasma cells, similar to issues encountered with CAR-T therapies [36–38]. Similarly, BITE therapies targeting CD38/CD3, such as AMG 424, have shown strong MM cell eradication but carry a risk of ‘off-tumor’ T-cell cytotoxicity, potentially impacting normal B, T, and NK cells [39]. To address the toxicities and limited biodistribution linked with the continuous infusion of recombinant BITE proteins, we propose engineering T cells to express and secrete these proteins. This approach removes the need for constant infusion, potentially improving treatment efficacy.

This study investigates engineered T cells secreting bispecific engager molecules targeting B7-H3 (αB7-H3-αCD3 ENG T cells), an antigen upregulated in MM. These molecules bind specifically to CD3 on T cells and B7-H3 on MM cells, bringing T cells close to MM cells and activating them to release cytotoxic cytokines and proliferate. This approach induces tumor cytotoxicity and redirects bystander T cells to target B7-H3-positive tumor cells, demonstrating potent antitumor activity and bypassing immune evasion [40]. Significant progress has been made in the fight against CD19-positive malignancies with T cell redirection strategies, such as CAR-T cells [41] and BITEs [18, 22]. While traditional therapies like CAR T cells and BITEs face challenges such as prolonged infusion times and limited biodistribution, [22, 24], ENG T cells offer a promising solution [25, 26]. This novel platform, showing potential in various cancer models, could revolutionize cancer treatment, especially for MM. Current FDA-approved BCMA-targeting therapies for MM, like CAR-T therapies and BITEs, require extensive prior treatments before administration. Despite initial successes, relapses are common, and some patients may develop alterations in the BCMA gene that affect BITE binding [42]. Therefore, our alternative approach using ENG-T cells targeting the B7-H3 antigen was developed to address these challenges.

B7-H3 (CD276), a member of the suppressive B7 family checkpoint molecules, is widely expressed in various human cancers and plays a crucial role in tumor progression [43]. The B7-H3 expression in MM is relatively low compared to other cancers but is still present in a subset of cases [44]. Studies have shown variable expression rates, generally ranging from 15 to 30% in MM cells. This expression is significant as B7-H3 plays a role in immune evasion, making it a potential target for immunotherapy in MM [45, 46]. Lin et al. discovered that B7-H3 promotes drug resistance, growth, and glycolysis in MM cells through the JAK2/STAT3 and PI3K/AKT signaling pathways. Their study also revealed that B7-H3 induces the oxidation of Src and the negative regulator SHP-1, leading to the phosphorylation of STAT3 and AKT. This activation triggers E3-ubiquitin ligase c-Cbl, resulting in the proteasomal degradation of SOCS3, a key negative regulator of STAT3 [45]. Our research confirmed differential expression of B7-H3 in MM cells: NCI-H929 showed low expression, L-363 exhibited medium to high expression, and KMS-12-PE displayed high expression on the cell surface (Fig. 1). Although studies demonstrate efficacy in B7-H3^low^ cell lines, further analysis is needed to determine the therapeutic threshold for antigen expression to optimize patient response.

To generate αB7-H3-αCD3 ENG T cells, we used a lentivirus system to create a construct that transduces T cells to secrete αB7-H3-αCD3 BITE molecules (Fig. 2A). We utilized the humanized monoclonal antibody 8H9, targeting B7-H3, due to its advanced stage of approval, known as ^131^I-omburtamab [47, 48], and use in other antibody-based treatments, including BITEs and various CAR-T cell constructs.[28, 49, 50]. Preclinical models have shown the potential efficacy of αB7-H3 8H9 therapy against solid and hematologic tumors.

Using AlphaFold, we predicted the protein structure of αB7-H3-αCD3 BITE, showing accurate superimposition of the αB7-H3 scFv and αCD3 scFv on the target structure (Fig. 2B). We established a stable HEK293T system to produce and secrete αB7-H3-αCD3 BITE, evaluating its binding capacity with B7-H3-positive target cells (Fig. 3E) and CD3-positive cells (Fig. 3H). Our results demonstrated successful binding to B7-H3 molecules (Fig. 3F and G) and effective interaction with CD3-positive cells (Fig. 3I and J). Previous studies have shown that BITEs using the αCD3 scFv from the OKT3 monoclonal antibody effectively induce T cell activation and proliferation [51].

αB7-H3-αCD3 ENG T cells, generated from healthy donor T cells, displayed surface molecule expression (Fig. 4A, B), predominantly comprising CD8^+^ cytotoxic T cells (Fig. 4C, D) with central memory T cell subsets (Fig. 4E). Studies by Dirk H Busch [52] and Qingjun Liu [53] suggest that memory T cell phenotypes exhibit better persistence and anticancer effects in vivo. Therefore, the increased population of CD8^+^ cytotoxic T cells with memory phenotype in our transduced T cells secreting αB7-H3-αCD3 BITE molecules may enhance cytotoxic activity and persistence against MM cells in vivo. Three exhaustion markers—PD-1, LAG-3, and TIM-3—were evaluated in CD3^+^ cells using flow cytometry (Fig. 4F). PD-1 expression was upregulated in PHA-L activated T cells but remained low in both untransduced (UTD) T cells and αB7-H3-αCD3 ENG T cells. Compared to UTD T cells, the ENG T cells showed lower LAG-3 expression, while TIM-3 was similarly and significantly increased in both ENG and PHA-L activated T cells. These data indicate that the ENG T cells exhibit a modified exhaustion profile, with reduced PD-1 and LAG-3 but elevated TIM-3.

To evaluate the performance of αB7-H3-αCD3 ENG T cells, we conducted assays assessing their anti-tumor activity, proliferation, and cytokine response against different levels of B7-H3 expression in MM cell lines. We first examined their toxicity against SupT-1 (B7-H3^neg^) cells, comparing it to that of UTD T cells. Our findings revealed that αB7-H3-αCD3 ENG T cells exhibited no statistically significant cytotoxicity against SupT-1 cells, indicating their specificity (Fig. 5A). Notably, increasing the effector cell dose correlated with elevated cytotoxicity, likely due to HLA disparity between the healthy donor-derived effector cells and the target SupT-1 cells, potentially triggering immune cell attack [54, 55]. Remarkably, αB7-H3-αCD3 ENG T cells demonstrated superior efficacy in eliminating NCI-H929 (B7-H3^low^) and L-363 (B7-H3^medium^) target cells compared to UTD T cells, particularly at an E:T ratio of 5:1 (Fig. 5B, C). Furthermore, at an E:T ratio of 1:1, αB7-H3-αCD3 ENG T cells outperformed UTD T cells in eradicating high B7-H3 expression (KMS-12-PE) cells (Fig. 5D). These ENG T cells also displayed a trend toward heightened proliferation, especially when co-cultured with the L-363 (B7-H3^medium^) and KMS-12-PE (B7-H3^high^) cell lines (Fig. 6). Our investigation underscores the significant potential of αB7-H3-αCD3 ENG T cells in combatting cancer. Through in vitro assessments across various cell lines, we observed their robust anti-tumor effects and ability to enhance T cell proliferation and activation, contingent upon B7-H3 expression levels. Consistent with Yin et al*.*'s findings [56], T cell-secreted 806BITE exhibits greater sensitivity than 806CAR T cells, suggesting a promising approach for targeting tumors with low or variable antigen expression levels. While prior research has not yet produced T cells secreting BITE molecules targeting both B7-H3 and CD3, comparisons with similar structures targeting different antigens yielded analogous outcomes, affirming their efficacy in precisely and efficiently destroying target cells [25, 26, 57]. Minimizing on-target off-tumor toxicity is crucial for the safety of ENG T cell therapy. Our findings indicate that αB7-H3-αCD3 ENG T cells are likely to exhibit minimal off-target toxicity, as B7-H3 expression is largely restricted to a few normal tissues. Consequently, these ENG T cells predominantly target B7-H3-positive cancer cells rather than affecting normal tissues [28, 29]. Supporting this, Grote et al*.* [58] showed that B7-H3 CAR-NK-92 cells effectively attacked B7-H3-positive neuroblastoma cells with no off-target toxicity, both in vitro and in vivo. Similarly, Liu et al*.* [49] reported that B7-H3/CD16 BITE cells enhanced cytotoxicity against B7-H3-positive solid tumors without causing off-target effects. Together, these findings suggest that the localized activity of αB7-H3-αCD3 ENG T cells within tumors may reduce systemic toxicity by limiting cross-reactivity in other organs.

Key indicators of cytotoxic activity and cytokine production, including IL-2, TNF-α, sFas, IFN-γ, granzyme A, granzyme B, perforin, and granulysin in the culture media of αB7-H3-αCD3 ENG T cells co-cultured with NCI-H929 (B7-H3^low^), L-363(B7-H3^medium^), and KMS-12-PE (B7-H3^high^) cells, were significantly upregulated compared to UTD T cells (Fig. 7). IL-2, a vital T cell growth factor, is a critical activation factor for T and NK cell proliferation and differentiation. Upon target cell killing by cytotoxic T lymphocytes, granules containing perforin and granzymes are released [59]. TNF-α and IFN-γ play roles in tumor cell killing and contribute to T cell-induced bystander tumor cell lysis by upregulating ICAM-1 and Fas [60]. Importantly, IFN-γ also significantly enhances daratumumab-mediated cytotoxicity towards tumor cells [61]. Our research suggests that αB7-H3-αCD3 ENG T cells activate T cells, inducing tumor cell killing. The αB7-H3-αCD3 BITE comprises two scFvs: one recognizes the tumor antigen, B7-H3, and the other identifies the CD3 molecule on T cells, leading to cytotoxicity, which can then be induced via bystander T cells. To validate this hypothesis, we tested whether αB7-H3-αCD3 BITE could redirect PHA-L activated T cells or bystander T cells to kill B7-H3-expressing MM cell lines. The results indicated that the combination of αB7-H3-αCD3 ENG T cell supernatant with PHA-L activated T cells was highly effective in killing B7-H3-expressing MM cells, including NCI-H929, L-363, and KMS-12-PE, in a dose-dependent manner compared to PHA-L activated T cells without αB7-H3-αCD3 ENG T cell supernatant (Fig. 8). Previous studies have shown that leukemia or lymphoma patients treated with CD19-targeted BITE (blinatumomab) achieved sustained remission [18, 23, 62]; however, repeated injections were necessary due to the short half-life of BITEs in circulation [63]. CAR-BITE T cells and BITE T cells initially showed superior control of tumor growth in a glioma mouse model compared to CAR T cells, but their effectiveness waned 20 days. While BITE T cells demonstrate comparable proliferation capacity to CAR-T cells in vitro, concerns remain regarding their long-term persistence because stimulation through BITEs alone upregulated the expression of several immune checkpoint inhibitors, ultimately leading to T cell exhaustion and impaired proliferation [64]. The expression of PD-1, LAG-3, and TIM-3 during extended co-culture, compared to conventional CAR-T cell therapies, should be investigated. It is crucial to determine whether the exhaustion phenotype is induced by this activation or if it is an inherent characteristic of the therapy itself. Although promising in cancer therapy, further research is imperative to address long-term efficacy concerns. Several reports suggest that co-stimulatory signals such as 4-1BB and CD28 can enhance T-cell activation by BITEs, potentially aiding in maintaining their function and integrating the benefits of BITE and CAR-T cell therapies [65, 66].

Our study does not include an in-depth analysis of T cell exhaustion over time or the effects of additional checkpoint inhibitors like TIGIT. However, we recognize that these factors are crucial for a comprehensive understanding of immune regulation. Further studies should examine these elements and investigate strategies to mitigate T cell exhaustion during prolonged BITE secretion, which could enhance the therapeutic potential of this approach. Additionally, the limited bystander T cells in NSG mice could explain the ineffectiveness of BITE therapy, as unmodified T cells may not survive beyond 20 days post-administration due to insufficient stimulation [67]. Our findings suggest that the use of αB7-H3-αCD3 ENG T cells may help address these issues, potentially controlling tumor growth and providing long-term anti-tumor effects effectively. Future studies that incorporate 1:1:1 co-cultures of UTD T cells, ENG T cells, and tumor cells would provide valuable insights into the bystander effect, potentially highlighting the advantages of this therapeutic approach over conventional BITE therapies. Such analyses could improve our understanding of how UTD T cells contribute to the overall antitumor response, clarifying their role in prolonging treatment efficacy. Combining αB7-H3-αCD3 ENG T cells with multi-specific antibodies targeting antigens commonly found on MM cells—such as BCMA, CD38, and CD138—could further enhance treatment efficacy, though this hypothesis requires validation through in vitro and in vivo studies using MM cancer xenograft mouse models. Additionally, it is important to investigate the molecular and cellular events following infusion, including changes in the tumor microenvironment, immune cell interactions, cytokine signaling, and potential resistance mechanisms. Understanding these factors will be essential for optimizing engineered-T cell therapies and developing strategies to reduce treatment-related toxicities.

It is important to acknowledge the limitations of our study. Our analysis relies on a limited selection of MM cell lines, some of which express relatively low levels of antigens. According to the Human Protein Atlas, these cell lines are not the best representatives of the high B7-H3 expression typically seen in MM. Due to access constraints, expanding the range of cell lines or including primary MM cells would help confirm the generalizability of our findings. While the in vitro results are promising, the lack of in vivo validation is a significant limitation. Additionally, the study does not explore the role of the tumor microenvironment, which is known to influence immunotherapy resistance. Conducting preclinical animal studies would provide valuable insights into the therapy's safety, efficacy, and potential immune-related toxicities.

## Conclusion

In conclusion, our study demonstrates the efficacy of αB7-H3-αCD3 ENG T cells in targeting B7-H3-positive multiple myeloma (MM), highlighting their remarkable sensitivity and specificity. T cells producing αB7-H3-αCD3 BITE molecules show great promise for treating B7-H3-positive MM, representing significant progress in targeted T-cell therapies. These findings pave the way for further research and development of precise and potent immunotherapeutic strategies against this malignancy.

## Supplementary Information

Additional file1: Fig. S1. BITE protein concentration in culture supernatants, assessed through semiquantitative immunoblotting analysis and compared to known A20/αCD3 BITE concentrations. Immunoblotting utilizing an anti-c-Myc tag antibody identified A20/αCD3 BITE, and immunoblotting identified αB7-H3-αCD3 BITE proteins in the culture supernatants of stable cells. Densitometry data of A20/αCD3 BITE protein bands relative to GAPDH bands on SDS-PAGE were analyzed using ImageJ software. A standard curve was generated from the densitometry data. The concentration of αB7-H3-αCD3 BITE protein in culture supernatants was calculated from the A20/αCD3 BITE standard curve, using the densitometry data of αB7-H3-αCD3-BITE protein bands relative to GAPDH bands on SDS-PAGE, also analyzed via ImageJ software.

Additional file 2: Table 1. Cytokine production profiles of untransduced T cells and αB7-H3-αCD3 ENG T cells following co-culture with the SupT-1 cell line. Table 2. Cytokine production profiles of untransduced T cells and αB7-H3-αCD3 ENG T cells following co-culture with the NCI-H929cell line. Table 3. Cytokine production profiles of untransduced T cells and αB7-H3-αCD3 ENG T cells following co-culture with the L-363 cell line. Table 4. Cytokine production profiles of untransduced T cells and αB7-H3-αCD3 ENG T cells following co-culture with the KMS-12-PE cell line.

## Data Availability

Data are included in the manuscript.

## Abbreviations

ADCC: Antibody-dependent cellular cytotoxicity
ANOVA: Analysis of variance
ASCT: Autologous stem cell transplantation
ATCC: American Type Culture Collection
BCMA: B-cell maturation antigen
BITE: Bispecific T-cell engager
B7-H3: B7 homolog 3 protein
CAR T-cell: Chimeric antigen receptor T-cell
cilta-cel: Ciltacabtagene autoleucel
CRS: Cytokine release syndrome
CTFR: Cell Trace Far Red™ dye
ENG T cell: T cells secrete bispecific engager molecule
E:T ratios: Effector-to-target ratios
FBS: Fetal bovine serum
GAPDH: Glyceraldehyde 3-phosphate dehydrogenase
HEK293T: Human embryonic kidney 293 T
ide-cel: Idecabtagene vicleucel
ImiDs: Immunomodulatory drugs
mAbs: Monoclonal antibodies
MM: Multiple myeloma
PHA-L: Phytohemagglutinin-L
PIs: Proteasome inhibitors
pLDDT: Predicted local distance difference test
RRMM: Relapsed/refractory MM
scFv: Single-chain variable fragment
SDS-PAGE: Sodium dodecyl sulfate–polyacrylamide gel electrophoresis
SEM: The standard error of the mean
UTD T: Un-transduced T cells

## References

[1] Kumar SK, Rajkumar V, Kyle RA, et al. Multiple myeloma. Nat Rev Dis Primers. 2017;3:17046. 10.1038/nrdp.2017.46. 28726797 10.1038/nrdp.2017.46

[2] Padala SA, Barsouk A, Barsouk A, et al. Epidemiology, staging, and management of multiple myeloma. Med Sci. 2021;9(1):3. 10.3390/medsci9010003. 10.3390/medsci9010003 PMC7838784 33498356

[3] Rajkumar SV. Multiple myeloma: 2022 update on diagnosis, risk stratification, and management. Am J Hematol. 2022;97(8):1086–107. 10.1002/ajh.26590. 35560063 10.1002/ajh.26590 PMC9387011

[4] Dimopoulos MA, Oriol A, Nahi H, et al. Daratumumab, lenalidomide, and dexamethasone for multiple myeloma. N Engl J Med. 2016;375(14):1319–31. 10.1056/NEJMoa1607751. 27705267 10.1056/NEJMoa1607751

[5] Davis LN, Sherbenou DW. Emerging therapeutic strategies to overcome drug resistance in multiple myeloma. Cancers. 2021;13(7):1686. 10.3390/cancers13071686. 33918370 10.3390/cancers13071686 PMC8038312

[6] Brigle K, Rogers B. Pathobiology and diagnosis of multiple myeloma. Semin Oncol Nurs. 2017;33(3):225–36. 10.1016/j.soncn.2017.05.012. 28688533 10.1016/j.soncn.2017.05.012

[7] de Weers M, Tai Y-T, van der Veer MS, et al. Daratumumab, a novel therapeutic human CD38 monoclonal antibody, induces killing of multiple myeloma and other hematological tumors. J Immunol. 2011;186(3):1840–8. 10.4049/jimmunol.1003032. 21187443 10.4049/jimmunol.1003032

[8] Balasa B, Yun R, Belmar NA, et al. Elotuzumab enhances natural killer cell activation and myeloma cell killing through interleukin-2 and TNF-alpha pathways. Cancer Immunol Immunother. 2015;64(1):61–73. 10.1007/s00262-014-1610-3. 25287778 10.1007/s00262-014-1610-3 PMC4282702

[9] Raje N, Berdeja J, Lin Y, et al. Anti-BCMA CAR T-cell therapy bb2121 in relapsed or refractory multiple myeloma. N Engl J Med. 2019;380(18):1726–37. 10.1056/NEJMoa1817226. 31042825 10.1056/NEJMoa1817226 PMC8202968

[10] Martin T, Usmani SZ, Berdeja JG, et al. Ciltacabtagene autoleucel, an anti-B-cell maturation antigen chimeric antigen receptor T-cell therapy, for relapsed/refractory multiple myeloma: CARTITUDE-1 2-year follow-up. J Clin Oncol. 2023;41(6):1265–74. 10.1200/JCO.22.00842. 35658469 10.1200/JCO.22.00842 PMC9937098

[11] Munshi NC, Anderson LD Jr, Shah N, et al. Idecabtagene vicleucel in relapsed and refractory multiple myeloma. N Engl J Med. 2021;384(8):705–16. 10.1056/NEJMoa2024850. 33626253 10.1056/NEJMoa2024850

[12] Berdeja JG, Madduri D, Usmani SZ, et al. Ciltacabtagene autoleucel, a B-cell maturation antigen-directed chimeric antigen receptor T-cell therapy in patients with relapsed or refractory multiple myeloma (CARTITUDE-1): a phase 1b/2 open-label study. Lancet. 2021;398(10297):314–24. 10.1016/S0140-6736(21)00933-8. 34175021 10.1016/S0140-6736(21)00933-8

[13] Roex G, Timmers M, Wouters K, et al. Safety and clinical efficacy of BCMA CAR-T-cell therapy in multiple myeloma. J Hematol Oncol. 2020;13(1):164. 10.1186/s13045-020-01001-1. 33272302 10.1186/s13045-020-01001-1 PMC7713173

[14] Wu JF, Dhakal B. BCMA-targeted CAR-T cell therapies in relapsed and/or refractory multiple myeloma: latest updates from 2023 ASCO Annual Meeting. J Hematol Oncol. 2023;16(1):86. 10.1186/s13045-023-01479-5. 37507805 10.1186/s13045-023-01479-5 PMC10385907

[15] Uckun FM. Overcoming the immunosuppressive tumor microenvironment in multiple myeloma. Cancers. 2021;13(9):2018. 10.3390/cancers13092018. 33922005 10.3390/cancers13092018 PMC8122391

[16] Tang L, Huang Z, Mei H, Hu Y. Immunotherapy in hematologic malignancies: achievements, challenges and future prospects. Signal Transduct Target Ther. 2023;8(1):306. 10.1038/s41392-023-01521-5. 37591844 10.1038/s41392-023-01521-5 PMC10435569

[17] Tian Z, Liu M, Zhang Y, Wang X. Bispecific T cell engagers: an emerging therapy for management of hematologic malignancies. J Hematol Oncol. 2021;14(1):75. 10.1186/s13045-021-01084-4. 33941237 10.1186/s13045-021-01084-4 PMC8091790

[18] Kantarjian H, Stein A, Gokbuget N, et al. Blinatumomab versus chemotherapy for advanced acute lymphoblastic leukemia. N Engl J Med. 2017;376(9):836–47. 10.1056/NEJMoa1609783. 28249141 10.1056/NEJMoa1609783 PMC5881572

[19] Lopedote P, Shadman M. Targeted treatment of relapsed or refractory follicular lymphoma: focus on the therapeutic potential of mosunetuzumab. Cancer Manag Res. 2023;15:257–64. 10.2147/CMAR.S381493. 36941881 10.2147/CMAR.S381493 PMC10024536

[20] Davis JA, Granger K, Sakowski A, et al. Dual target dilemma: navigating epcoritamab vs. glofitamab in relapsed refractory diffuse large B-cell lymphoma. Expert Rev Hematol. 2023;16(12):915–8. 10.1080/17474086.2023.2285978. 37982732 10.1080/17474086.2023.2285978

[21] Pan D, Richter J. Teclistamab for multiple myeloma: clinical insights and practical considerations for a first-in-class bispecific antibody. Cancer Manag Res. 2023;15:741–51. 10.2147/CMAR.S372237. 37497430 10.2147/CMAR.S372237 PMC10368105

[22] Zhu M, Wu B, Brandl C, et al. Blinatumomab, a bispecific T-cell engager (BiTE®) for CD-19 targeted cancer immunotherapy: clinical pharmacology and its implications. Clin Pharmacokinet. 2016;55(10):1271–88. 10.1007/s40262-016-0405-4. 27209293 10.1007/s40262-016-0405-4

[23] Newman MJ, Benani DJ. A review of blinatumomab, a novel immunotherapy. J Oncol Pharm Pract. 2016;22(4):639–45. 10.1177/1078155215618770. 26607163 10.1177/1078155215618770

[24] Frankel SR, Baeuerle PA. Targeting T cells to tumor cells using bispecific antibodies. Curr Opin Chem Biol. 2013;17(3):385–92. 10.1016/j.cbpa.2013.03.029. 23623807 10.1016/j.cbpa.2013.03.029

[25] Iwahori K, Kakarla S, Velasquez MP, et al. Engager T cells: a new class of antigen-specific T cells that redirect bystander T cells. Mol Ther. 2015;23(1):171–8. 10.1038/mt.2014.156. 25142939 10.1038/mt.2014.156 PMC4426792

[26] Sangsuwannukul T, Supimon K, Chieochansin T, et al. Antitumor activity of T cells secreting αCD133-αCD3 bispecific T-cell engager against cholangiocarcinoma. PLoS ONE. 2022;17(3): e0265773. 10.1371/journal.pone.0265773. 35312724 10.1371/journal.pone.0265773 PMC8936442

[27] Kontos F, Michelakos T, Kurokawa T, et al. B7–H3: an attractive target for antibody-based immunotherapy. Clin Cancer Res. 2021;27(5):1227–35. 10.1158/1078-0432.CCR-20-2584. 33051306 10.1158/1078-0432.CCR-20-2584 PMC7925343

[28] Zhang Z, Jiang C, Liu Z, et al. B7–H3-targeted CAR-T cells exhibit potent antitumor effects on hematologic and solid tumors. Mol Ther Oncolytics. 2020;17:180–9. 10.1016/j.omto.2020.03.019. 32346608 10.1016/j.omto.2020.03.019 PMC7178328

[29] Zhang LY, Jin Y, Xia PH, et al. Integrated analysis reveals distinct molecular, clinical, and immunological features of B7–H3 in acute myeloid leukemia. Cancer Med. 2021;10(21):7831–46. 10.1002/cam4.4284. 34562306 10.1002/cam4.4284 PMC8559480

[30] Pant A, Medikonda R, Lim M. Alternative checkpoints as targets for immunotherapy. Curr Oncol Rep. 2020;22(12):126. 10.1007/s11912-020-00983-y. 33141349 10.1007/s11912-020-00983-y PMC7608303

[31] Ahmed M, Cheng M, Zhao Q, et al. Humanized affinity-matured monoclonal antibody 8H9 has Potent antitumor activity and binds to FG loop of tumor antigen B7–H3. J Biol Chem. 2015;290(50):30018–29. 10.1074/jbc.M115.679852. 26487718 10.1074/jbc.M115.679852 PMC4705981

[32] Suwanchiwasiri K, Phanthaphol N, Somboonpatarakun C, et al. Bispecific T cell engager-armed T cells targeting integrin alphanubeta6 exhibit enhanced T cell redirection and antitumor activity in cholangiocarcinoma. Biomed Pharmacother. 2024;175: 116718. 10.1016/j.biopha.2024.116718. 38744221 10.1016/j.biopha.2024.116718

[33] Jumper J, Evans R, Pritzel A, et al. Highly accurate protein structure prediction with AlphaFold. Nature. 2021;596(7873):583–9. 10.1038/s41586-021-03819-2. 34265844 10.1038/s41586-021-03819-2 PMC8371605

[34] Mariani V, Biasini M, Barbato A, Schwede T. lDDT: a local superposition-free score for comparing protein structures and models using distance difference tests. Bioinformatics. 2013;29(21):2722–8. 10.1093/bioinformatics/btt473. 23986568 10.1093/bioinformatics/btt473 PMC3799472

[35] Cho SF, Lin L, Xing L, et al. The immunomodulatory drugs lenalidomide and pomalidomide enhance the potency of AMG 701 in multiple myeloma preclinical models. Blood Adv. 2020;4(17):4195–207. 10.1182/bloodadvances.2020002524. 32898244 10.1182/bloodadvances.2020002524 PMC7479960

[36] Samur MK, Fulciniti M, Aktas Samur A, et al. Biallelic loss of BCMA as a resistance mechanism to CAR T cell therapy in a patient with multiple myeloma. Nat Commun. 2021;12(1):868. 10.1038/s41467-021-21177-5. 33558511 10.1038/s41467-021-21177-5 PMC7870932

[37] Da Via MC, Dietrich O, Truger M, et al. Homozygous BCMA gene deletion in response to anti-BCMA CAR T cells in a patient with multiple myeloma. Nat Med. 2021;27(4):616–9. 10.1038/s41591-021-01245-5. 33619368 10.1038/s41591-021-01245-5

[38] Pont MJ, Hill T, Cole GO, et al. γ-Secretase inhibition increases efficacy of BCMA-specific chimeric antigen receptor T cells in multiple myeloma. Blood. 2019;134(19):1585–97. 10.1182/blood.2019000050. 31558469 10.1182/blood.2019000050 PMC6871311

[39] Zuch de Zafra CL, Fajardo F, Zhong W, et al. Targeting multiple myeloma with AMG 424, a novel anti-CD38/CD3 bispecific T-cell-recruiting antibody optimized for cytotoxicity and cytokine release. Clin Cancer Res. 2019;25(13):3921–33. 10.1158/1078-0432.CCR-18-2752. 30918018 10.1158/1078-0432.CCR-18-2752

[40] Rendo MJ, Joseph JJ, Phan LM, DeStefano CB. CAR T-cell therapy for patients with multiple myeloma: current evidence and challenges. Blood Lymphat Cancer. 2022;12:119–36. 10.2147/BLCTT.S327016. 36060553 10.2147/BLCTT.S327016 PMC9439649

[41] June CH, Sadelain M. Chimeric antigen receptor therapy. N Engl J Med. 2018;379(1):64–73. 10.1056/NEJMra1706169. 29972754 10.1056/NEJMra1706169 PMC7433347

[42] Lee H, Ahn S, Maity R, et al. Mechanisms of antigen escape from BCMA- or GPRC5D-targeted immunotherapies in multiple myeloma. Nat Med. 2023;29(9):2295–306. 10.1038/s41591-023-02491-5. 37653344 10.1038/s41591-023-02491-5 PMC10504087

[43] Zhao B, Li H, Xia Y, et al. Immune checkpoint of B7–H3 in cancer: from immunology to clinical immunotherapy. J Hematol Oncol. 2022;15(1):153. 10.1186/s13045-022-01364-7. 36284349 10.1186/s13045-022-01364-7 PMC9597993

[44] Koumprentziotis IA, Theocharopoulos C, Foteinou D, et al. New emerging targets in cancer immunotherapy: the role of B7–H3. Vaccines (Basel). 2024;12(1):54. 10.3390/vaccines12010054. 38250867 10.3390/vaccines12010054 PMC10820813

[45] Lin L, Cao L, Liu Y, et al. B7–H3 promotes multiple myeloma cell survival and proliferation by ROS-dependent activation of Src/STAT3 and c-Cbl-mediated degradation of SOCS3. Leukemia. 2019;33(6):1475–86. 10.1038/s41375-018-0331-6. 30573782 10.1038/s41375-018-0331-6

[46] Picarda E, Ohaegbulam KC, Zang X. Molecular pathways: targeting B7–H3 (CD276) for human cancer immunotherapy. Clin Cancer Res. 2016;22(14):3425–31. 10.1158/1078-0432.CCR-15-2428. 27208063 10.1158/1078-0432.CCR-15-2428 PMC4947428

[47] 131I-omburtamab neuroblastoma with central nervous system/leptomeningeal metastases. Y-mAbs Therapeutics Inc. 2022. www.fda.gov/media/162617/download. Accessed 27 Feb 2024.

[48] FDA Briefing Document 131I-Omburtamab. Y-mAbs Therapeutics Inc. 2022. www.fda.gov/media/162614/download. Accessed 27 Feb 2024.

[49] Liu J, Yang S, Cao B, et al. Targeting B7–H3 via chimeric antigen receptor T cells and bispecific killer cell engagers augments antitumor response of cytotoxic lymphocytes. J Hematol Oncol. 2021;14(1):21. 10.1186/s13045-020-01024-8. 33514401 10.1186/s13045-020-01024-8 PMC7844995

[50] Birley K, Leboreiro-Babe C, Rota EM, et al. A novel anti-B7-H3 chimeric antigen receptor from a single-chain antibody library for immunotherapy of solid cancers. Mol Ther Oncolytics. 2022;26:429–43. 10.1016/j.omto.2022.08.008. 36159778 10.1016/j.omto.2022.08.008 PMC9467911

[51] Horn LA, Ciavattone NG, Atkinson R, et al. CD3xPDL1bi-specific T cell engager( BiTE) simultaneously activates T cells and NK T cells, kills PDL1(+) tumor cells, and extends the survival of tumor-bearing humanized mice. Oncotarget. 2017;8:57964–80. 10.18632/oncotarget.19865. 28938530 10.18632/oncotarget.19865 PMC5601626

[52] Busch DH, Frassle SP, Sommermeyer D, Buchholz VR, Riddell SR. Role of memory T cell subsets for adoptive immunotherapy. Semin Immunol. 2016;28(1):28–34. 10.1016/j.smim.2016.02.001. 26976826 10.1016/j.smim.2016.02.001 PMC5027130

[53] Liu Q, Sun Z, Chen L. Memory T cells: strategies for optimizing tumor immunotherapy. Protein Cell. 2020;11(8):549–64. 10.1007/s13238-020-00707-9. 32221812 10.1007/s13238-020-00707-9 PMC7381543

[54] Rutten CE, van Luxemburg-Heijs SA, van der Meijden ED, et al. HLA-DPB1 mismatching results in the generation of a full repertoire of HLA-DPB1-specific CD4+ T cell responses showing immunogenicity of all HLA-DPB1 alleles. Biol Blood Marrow Transplant. 2010;16(9):1282–92. 10.1016/j.bbmt.2010.03.018. 20350610 10.1016/j.bbmt.2010.03.018

[55] Furst D, Neuchel C, Tsamadou C, Schrezenmeier H, Mytilineos J. HLA matching in unrelated stem cell transplantation up to date. Transfus Med Hemother. 2019;46(5):326–36. 10.1159/000502263. 31832058 10.1159/000502263 PMC6876603

[56] Yin Y, Rodriguez JL, Li N, et al. Locally secreted BiTEs complement CAR T cells by enhancing killing of antigen heterogeneous solid tumors. Mol Ther. 2022;30(7):2537–53. 10.1016/j.ymthe.2022.05.011. 35570396 10.1016/j.ymthe.2022.05.011 PMC9263323

[57] Vaidya A, Doherty E, Wu X, et al. Improving the anti-acute myeloid leukemia activity of CD123-specific engager T cells by MyD88 and CD40 costimulation. Haematologica. 2023;108(4):1039–52. 10.3324/haematol.2021.279301. 35899386 10.3324/haematol.2021.279301 PMC10071120

[58] Grote S, Chan KCH, Baden C, et al. CD276 as a novel CAR NK-92 therapeutic target for neuroblastoma. Adv Cell Gene Ther. 2020. 10.1002/acg2.105.

[59] Lins Ferreira V, Borba H, Bonetti A, Leonart L, Pontarolo R. Cytokines and interferons: types and functions.10.5772/intechopen.745502018. 10.5772/intechopen.74550 (Errata)

[60] Ross SL, Sherman M, McElroy PL, et al. Bispecific T cell engager (BiTE(R)) antibody constructs can mediate bystander tumor cell killing. PLoS ONE. 2017;12(8): e0183390. 10.1371/journal.pone.0183390. 28837681 10.1371/journal.pone.0183390 PMC5570333

[61] Fatehchand K, McMichael EL, Reader BF, et al. Interferon-gamma promotes antibody-mediated fratricide of acute myeloid leukemia cells. J Biol Chem. 2016;291(49):25656–66. 10.1074/jbc.M116.753145. 27780867 10.1074/jbc.M116.753145 PMC5207262

[62] Dufner V, Sayehli CM, Chatterjee M, et al. Long-term outcome of patients with relapsed/refractory B-cell non-Hodgkin lymphoma treated with blinatumomab. Blood Adv. 2019;3(16):2491–8. 10.1182/bloodadvances.2019000025. 31451445 10.1182/bloodadvances.2019000025 PMC6712531

[63] Goebeler ME, Bargou RC. T cell-engaging therapies - BiTEs and beyond. Nat Rev Clin Oncol. 2020;17(7):418–34. 10.1038/s41571-020-0347-5. 32242094 10.1038/s41571-020-0347-5

[64] Choi BD, Yu X, Castano AP, et al. CAR-T cells secreting BiTEs circumvent antigen escape without detectable toxicity. Nat Biotechnol. 2019;37(9):1049–58. 10.1038/s41587-019-0192-1. 31332324 10.1038/s41587-019-0192-1

[65] Correnti CE, Laszlo GS, de van der Schueren WJ, et al. Simultaneous multiple interaction T-cell engaging (SMITE) bispecific antibodies overcome bispecific T-cell engager (BiTE) resistance via CD28 co-stimulation. Leukemia. 2018;32(5):1239–43. 10.1038/s41375-018-0014-3. 29588544 10.1038/s41375-018-0014-3 PMC5943151

[66] Claus C, Ferrara C, Xu W, et al. Tumor-targeted 4–1BB agonists for combination with T cell bispecific antibodies as off-the-shelf therapy. Sci Transl Med. 2019. 10.1126/scitranslmed.aav5989. 31189721 10.1126/scitranslmed.aav5989 PMC7181714

[67] Hegde PS, Chen DS. Top 10 challenges in cancer immunotherapy. Immunity. 2020;52(1):17–35. 10.1016/j.immuni.2019.12.011. 31940268 10.1016/j.immuni.2019.12.011

