# Supplementary material for: Engineered T cells secreting αB7-H3-αCD3 bispecific engagers for enhanced anti-tumor activity against B7-H3 positive multiple myeloma: a novel therapeutic approach
Source: J Transl Med. 2025 Jan 13;23:54. doi: 10.1186/s12967-024-05923-z (PMC11727291; doi:10.1186/s12967-024-05923-z)
Supplement: Supplementary file 1 — Additional file1: Fig. S1. BITE protein concentration in culture supernatants, assessed through semiquantitative immunoblotting analysis and compared to known A20/αCD3 BITE concentrations. Immunoblotting utilizing an anti-c-Myc tag antibody identified A20/αCD3 BITE, and immunoblotting identified αB7-H3-αCD3 BITE proteins in the culture supernatants of stable cells. Densitometry data of A20/αCD3 BITE protein bands relative to GAPDH bands on SDS-PAGE were analyzed using ImageJ software. A standard curve was generated from the densitometry data. The concentration of αB7-H3-αCD3 BITE protein in culture supernatants was calculated from the A20/αCD3 BITE standard curve, using the densitometry data of αB7-H3-αCD3-BITE protein bands relative to GAPDH bands on SDS-PAGE, also analyzed via ImageJ software. [file 12967_2024_5923_MOESM1_ESM.pdf]

# Engineered T Cells Secreting αB7-H3-αCD3 Bispecific Engagers for Enhanced Anti-Tumor Activity Against B7-H3 Positive Multiple Myeloma: A Novel Therapeutic Approach

Punchita Rujirachaivej[1#], Teerapong Siriboonpiputtana[2#], Kornkan Choomee[3,4], Kamonlapat Supimon[3,4], Thanich Sangsuwannukul[5], Pucharee Songprakhon[3,4], Krissada Natungnuy[3,4], Piriya Luangwattananun[3,4], Pornpimon Yuti[3,4], Mutita Junking[3,4*], Pa-thai Yenchitsomanus[3,4*]

[1]Graduate Program in Clinical Pathology, Department of Pathology, Faculty of Medicine Ramathibodi Hospital, Mahidol University, Bangkok, Thailand

[2]Department of Pathology, Faculty of Medicine Ramathibodi Hospital, Mahidol University, Bangkok, Thailand

[3]Siriraj Center of Research Excellence for Cancer Immunotherapy (SiCORE-CIT), Faculty of Medicine Siriraj Hospital, Mahidol University, Bangkok, Thailand

[4]Division of Molecular Medicine, Research Department, Faculty of Medicine Siriraj Hospital, Mahidol University, Bangkok, Thailand

[5]Department of Molecular Medicine, Mayo Clinic Rochester, MN USA

[#] These two authors contributed equally to this work.

[*]Corresponding authors: Mutita Junking (E-mail: mjunking@gmail.com; mutita.jun@mahidol.ac.th), Pa-thai Yenchitsomanus (E-mail: ptyench@gmail.com; pathai.yen@mahidol.edu)

# Supplementary Information

A

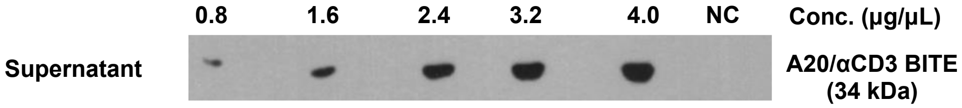


B

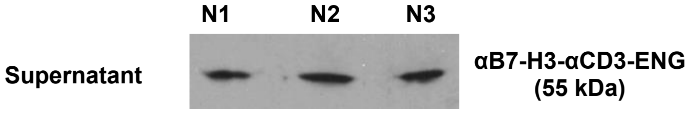


C

| Conc. A20 A20/αCD3 BITE (µg/µL) | Densitometry using ImageJ |
|---|---|
| NC | 94.9 |
| 0.8 | 2062 |
| 1.6 | 5305.0 |
| 2.4 | 11680.3 |
| 3.2 | 14703.8 |
| 4.0 | 15570.5 |

D

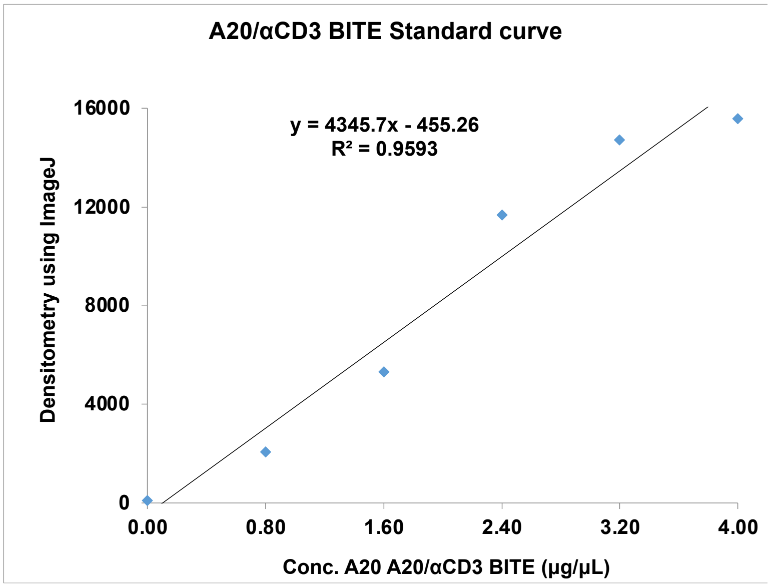


E

| αB7-H3-αCD3-ENG | Densitometry using ImageJ | Conc. αB7-H3-αCD3-ENG (μg/mL) |
|---|---|---|
| N1 | 7156.69 | 38.55 |
| N2 | 9200.93 | 50.31 |
| N3 | 8904.83 | 48.61 |
| Mean | 8420.82 | 45.82 |

**Fig.1 BITE protein concentration in culture supernatants, assessed through semiquantitative immunoblotting analysis and compared to known A20/αCD3 BITE concentrations.** Immunoblotting utilizing an anti-c-Myc tag antibody identified **(A)** A20/αCD3 BITE and **(B)** αB7-H3-αCD3 BITE proteins in the culture supernatants of stable cells. **(C)** Densitometry data of A20/αCD3 BITE protein bands relative to GAPDH bands on SDS-PAGE were analyzed using ImageJ software to generate **(D)** the standard curve. Subsequently, **(E)** the concentration of αB7-H3-αCD3 BITE protein in culture supernatants was calculated from the A20/αCD3 BITE standard curve, utilizing the densitometry data of αB7-H3-αCD3-BITE protein bands relative to GAPDH bands on SDS-PAGE, also analyzed via ImageJ software.
