# Supplementary material for: Engineered T cells secreting αB7-H3-αCD3 bispecific engagers for enhanced anti-tumor activity against B7-H3 positive multiple myeloma: a novel therapeutic approach
Source: J Transl Med. 2025 Jan 13;23:54. doi: 10.1186/s12967-024-05923-z (PMC11727291; doi:10.1186/s12967-024-05923-z)
Supplement: Supplementary file 2 — Additional file 2: Table 1. Cytokine production profiles of untransduced T cells and αB7-H3-αCD3 ENG T cells following co-culture with the SupT-1 cell line. Table 2. Cytokine production profiles of untransduced T cells and αB7-H3-αCD3 ENG T cells following co-culture with the NCI-H929cell line. Table 3. Cytokine production profiles of untransduced T cells and αB7-H3-αCD3 ENG T cells following co-culture with the L-363 cell line. Table 4. Cytokine production profiles of untransduced T cells and αB7-H3-αCD3 ENG T cells following co-culture with the KMS-12-PE cell line. [file 12967_2024_5923_MOESM2_ESM.pdf]

Engineered T Cells Secreting αB7-H3-αCD3 Bispecific Engagers for Enhanced Anti-Tumor Activity Against B7-H3 Positive Multiple Myeloma: A Novel Therapeutic Approach

Punchita Rujirachaivej[1#], Teerapong Siriboonpiputtana[2#], Kornkan Choomee[3,4], Kamonlapat Supimon[3,4], Thanich Sangsuwannukul[5], Pucharee Songprakhon[3,4], Krissada Natungnuy[3,4], Piriya Luangwattananun[3,4], Pornpimon Yuti[3,4], Mutita Junking[3,4*], Pa-thai Yenchitsomanus[3,4*]

[1]Graduate Program in Clinical Pathology, Department of Pathology, Faculty of Medicine Ramathibodi Hospital, Mahidol University, Bangkok, Thailand

[2]Department of Pathology, Faculty of Medicine Ramathibodi Hospital, Mahidol University, Bangkok, Thailand

[3]Siriraj Center of Research Excellence for Cancer Immunotherapy (SiCORE-CIT), Faculty of Medicine Siriraj Hospital, Mahidol University, Bangkok, Thailand

[4]Division of Molecular Medicine, Research Department, Faculty of Medicine Siriraj Hospital, Mahidol University, Bangkok, Thailand

[5]Department of Molecular Medicine, Mayo Clinic Rochester, MN USA

[#] These two authors contributed equally to this work.

[*]Corresponding authors: Mutita Junking (E-mail: mjunking@gmail.com; mutita.jun@mahidol.ac.th), Pa-thai Yenchitsomanus (E-mail: ptyench@gmail.com; pathai.yen@mahidol.edu)

Supplementary Information

Table 1. Cytokine production profiles of untransduced T cells and αB7-H3-αCD3 ENG T cells following co-culture with the with SupT-1 (B7-H3^{Neg}) cell line. Cytokine levels were assessed using the LEGENDplex^{TM} Human CD8/NK cell panel Cytokine Bead Array (CBA), measuring 13 cytokines and proteins after a 24 hours co-culture period.

**Cytokines**	**UTD T cells**	**αB7-H3-αCD3 ENG T cells**	
	**Mean ± SEM (pg/ml)**	**Mean ± SEM (pg/ml)**	***P****
**IL-2**	9.1 ± 2.2	8.5 ± 1.7	0.8220
**IL-4**	0.3 ± 0.1	0.3 ± 0.1	0.7341
**IL-6**	4.7 ± 0.5	32.7 ± 31.2	0.4051
**IL-10**	1.9 ± 0.3	2.6 ± 0.5	0.2522
**IL-17A**	2.3 ± 0.9	2.3 ± 0.5	0.9760
**TNF-α**	7.5 ± 3.6	8.4 ± 5.8	0.8933
**sFas**	9.0 ± 1.2	10.8 ± 2.3	0.5123
**sFasL**	60.9 ± 16.3	33.6 ± 11.2	0.2156
**IFN-γ**	926.7 ± 331.5	1363.1 ± 457.2	0.4690
**Granzyme A**	1329.6 ± 140.3	1838.9 ± 356.9	0.2325
**Granzyme B**	18582.0 ± 6110.3	25290.0 ± 5452.8	0.4439
**Perforin**	349.6 ± 81.1	333.6 ± 82.6	0.8947
**Granulysin**	2513.1 ± 635.8	2466.7 ± 990.5	0.9699

* The data were derived from 4 independent experiments (N=4), and the results are presented as mean ± standard error of the mean (SEM). Statistical significance was determined using unpaired Student's t-tests (*$p<0.05$, **$p<0.01$, ***$p<0.001$, and ****$p<0.0001$).

Table 2. Cytokine production profiles of untransduced T cells and αB7-H3-αCD3 ENG T cells following co-culture with the NCI-H929 (B7-H3^{Low}) cell line. Cytokine levels were assessed using the LEGENDplex™ Human CD8/NK cell panel Cytokine Bead Array (CBA), measuring 13 cytokines and proteins after a 24 hours co-culture period.

Cytokines	UTD T cells	αB7-H3-αCD3 ENG T cells	
	Mean ± SEM (pg/ml)	Mean ± SEM (pg/ml)	*P**
**IL-2**	2.9 ± 1.5	12.8 ± 1.0	0.0013
**IL-4**	0.3± 0.1	0.3 ± 0.0	0.6250
**IL-6**	5.0 ± 2.0	5.9 ± 1.8	0.7530
**IL-10**	6.5 ± 1.2	4.8 ± 2.2	0.5109
**IL-17A**	2.1± 0.9	2.3 ± 0.5	0.8014
**TNF-α**	3.4 ± 0.8	23.4 ± 4.8	0.0063
**sFas**	6.3 ± 0.7	11.3 ± 0.7	0.0021
**sFasL**	56.3 ± 6.5	43.2 ± 1.5	0.0980
**IFN-γ**	308.5 ± 72.1	2158.6 ± 489.3	0.0096
**Granzyme A**	361.0 ± 94.6	4835.6 ± 1584.1	0.0304
**Granzyme B**	6021.4 ± 1539.5	18352.2 ± 4268.4	0.0348
**Perforin**	261.7 ± 34.8	437.1 ± 27.4	0.0074
**Granulysin**	1065.3 ± 211.1	2795.6 ± 587.2	0.0323

* The data were gathered from 4 independent experiments (N=4), and the results are presented as mean ± standard error of the mean (SEM). Statistical significance was assessed using unpaired Student's t-tests (*p<0.05, **p<0.01, ***p<0.001, and ****p<0.0001).

Table 3. Cytokine production profiles of untransduced T cells and αB7-H3-αCD3 ENG T cells following co-culture with the L-363 (B7-H3^{Medium}) cell line. Cytokine levels were assessed using the LEGENDplex™ Human CD8/NK cell panel Cytokine Bead Array (CBA), measuring 13 cytokines and proteins after a 24 hour co-culture period.

Cytokines	UTD T cells	αB7-H3-αCD3 ENG T cells	
	Mean ± SEM (pg/ml)	Mean ± SEM (pg/ml)	***P****
**IL-2**	5.0 ± 2.8	346.8 ± 130.4	0.0395
**IL-4**	0.3 ± 0.1	0.2 ± 0.1	0.6922
**IL-6**	7.5 ± 7.0	3.7 ± 2.8	0.6370
**IL-10**	1.2 ± 0.3	1.3 ± 0.6	0.9224
**IL-17A**	2.3 ± 0.5	3.1 ± 0.9	0.4799
**TNF-α**	6.3 ± 3.6	30.5 ± 6.9	0.0205
**sFas**	7.7 ± 0.2	11.6 ± 1.0	0.0106
**sFasL**	85.5 ± 12.2	75.0 ± 4.7	0.4487
**IFN-γ**	603.4 ± 227.3	7508.0 ± 1852.6	0.0101
**Granzyme A**	397.9 ± 109.8	4606.3 ± 1520.0	0.0328
**Granzyme B**	4038.1 ± 1286.9	21877.4 ± 7174.4	0.0500
**Perforin**	157.6 ± 80.4	470.8 ± 43.5	0.0140
**Granulysin**	257.5 ± 85.1	4221.2 ± 967.3	0.0065

* The data were derived from 4 independent experiments (N=4), and the results are presented as mean ± standard error of the mean (SEM). Statistical significance was assessed using unpaired Student's t-tests (*p<0.05, **p<0.01, ***p<0.001, and ****p<0.0001).

Table 4. Cytokine production profiles of untransduced T cells and αB7-H3-αCD3 ENG T cells, in response following co-culture with the KMS-12-PE (B7-H3^{High}) cell line. Cytokine levels were quantified using the LEGENDplex^{TM} Human CD8/NK cell panel Cytokine Bead Array (CBA), measuring 13 cytokines and proteins after a 24 hour co-culture period.

**Cytokines**	**UTD T cells**	**αB7-H3-αCD3 ENG T cells**	
	**Mean ± SEM (pg/ml)**	**Mean ± SEM (pg/ml)**	***P****
**IL-2**	3.0 ± 1.2	316.8 ± 127.4	0.0489
**IL-4**	0.4 ± 0.1	0.7 ± 0.2	0.3697
**IL-6**	0.6 ± 0.3	2.7 ± 1.8	0.2727
**IL-10**	1.2 ± 0.1	1.9 ± 0.4	0.1564
**IL-17A**	1.2 ± 0.3	5.4 ± 1.4	0.0252
**TNF-α**	3.8 ± 3.4	68.0 ± 24.0	0.0381
**sFas**	9.5± 0.6	31.2 ± 7.3	0.0245
**sFasL**	76.7 ± 16.6	83.0 ± 3.4	0.7245
**IFN-γ**	1668.3 ± 349.3	7696.0 ± 1611.3	0.0106
**Granzyme A**	550.7 ± 192.4	7674.5 ± 1933.8	0.0105
**Granzyme B**	4225.0 ± 2655.5	20740.5 ± 3581.5	0.0100
**Perforin**	191.4 ± 39.6	746.7 ± 104.0	0.0025
**Granulysin**	389.7 ± 115.9	5122.7 ± 228.7	<0.0001

* The data were collected from 4 independent experiments (N=4), and the results are presented as mean ± standard error of the mean (SEM). Statistical significance was assessed using unpaired Student's t-tests (*$p<0.05$, **$p<0.01$, ***$p<0.001$, and ****$p<0.0001$).
